# Supplementary material for: Modelling NHS England 111 demand for primary care services: a discrete event simulation
Source: BMJ Open. 2023 Sep 6;13(9):e076203. doi: 10.1136/bmjopen-2023-076203 (PMC10496671; doi:10.1136/bmjopen-2023-076203)
Supplement: Author's manuscript [file bmjopen-2023-076203.draft_revisions.pdf]

BMJ Open is committed to open peer review. As part of this commitment we make the peer review history of every article we publish publicly available.

When an article is published we post the peer reviewers' comments and the authors' responses online. We also post the versions of the paper that were used during peer review. These are the versions that the peer review comments apply to.

The versions of the paper that follow are the versions that were submitted during the peer review process. They are not the versions of record or the final published versions. They should not be cited or distributed as the published version of this manuscript.

BMJ Open is an open access journal and the full, final, typeset and author-corrected version of record of the manuscript is available on our site with no access controls, subscription charges or pay-per-view fees (http://bmjopen.bmj.com).

If you have any questions on BMJ Open's open peer review process please email info.bmjopen@bmj.com

BMJ Open

Modelling NHS 111 demand for primary care services: a discrete event simulation

Journal:	*BMJ Open*
Manuscript ID	bmjopen-2023-076203
Article Type:	Original research
Date Submitted by the Author:	02-Jun-2023
Complete List of Authors:	Pilbery, Richard; Yorkshire Ambulance Service NHS Trust, Research and Development<br>Smith, Madeleine; NHS Devon<br>Green, Jonathan; University of Plymouth Faculty of Health<br>Chalk, Daniel; University of Exeter Medical School,<br>O'Keeffe, Colin; The University of Sheffield, School of Health and Related Research
Keywords:	Primary Care < Primary Health Care, Telemedicine < BIOTECHNOLOGY & BIOINFORMATICS, Health informatics < BIOTECHNOLOGY & BIOINFORMATICS

SCHOLARONE™
Manuscripts

I, the Submitting Author has the right to grant and does grant on behalf of all authors of the Work (as defined in the below author licence), an exclusive licence and/or a non-exclusive licence for contributions from authors who are: i) UK Crown employees; ii) where BMJ has agreed a CC-BY licence shall apply, and/or iii) in accordance with the terms applicable for US Federal Government officers or employees acting as part of their official duties; on a worldwide, perpetual, irrevocable, royalty-free basis to BMJ Publishing Group Ltd ("BMJ") its licensees and where the relevant Journal is co-owned by BMJ to the co-owners of the Journal, to publish the Work in this journal and any other BMJ products and to exploit all rights, as set out in our licence.

The Submitting Author accepts and understands that any supply made under these terms is made by BMJ to the Submitting Author unless you are acting as an employee on behalf of your employer or a postgraduate student of an affiliated institution which is paying any applicable article publishing charge ("APC") for Open Access articles. Where the Submitting Author wishes to make the Work available on an Open Access basis (and intends to pay the relevant APC), the terms of reuse of such Open Access shall be governed by a Creative Commons licence – details of these licences and which Creative Commons licence will apply to this Work are set out in our licence referred to above.

Other than as permitted in any relevant BMJ Author's Self Archiving Policies, I confirm this Work has not been accepted for publication elsewhere, is not being considered for publication elsewhere and does not duplicate material already published. I confirm all authors consent to publication of this Work and authorise the granting of this licence.

For peer review only

Modelling NHS 111 demand for primary care services: a discrete event simulation

Author information

Richard Pilbery (Corresponding author)

Research paramedic

Yorkshire Ambulance Service NHS Trust

Springhill, Brindley Way

Wakefield 41 Business Park

Wakefield

WF2 0XQ

ORCID: https://orcid.org/0000-0002-5797-9788

email: r.pilbery@nhs.net

Madeleine Smith

NHS Devon

Jonathan Green

University of Plymouth

ORCID: https://orcid.org/0000-0001-5738-7515

Dr. Daniel Chalk

University of Exeter

ORCID: https://orcid.org/0000-0002-4165-4364

Colin O'Keeffe

University of Sheffield

ORCID: https://orcid.org/0000-0002-4484-7401

Keywords: 111, Telephone triage, primary care, emergency care, discrete event simulation

For peer review only

ABSTRACT

Objectives

This feasibility study aimed to model *in-silico* the current healthcare system for patients triaged to a primary care disposition following a call to NHS 111, and determine the effect of reconfiguring the healthcare system to ensure a timely primary care service contact.

Design

Discrete event simulation.

Setting

Single English NHS 111 call centre in Yorkshire.

Participants

Callers registered with a Bradford general practitioner (GP) who contacted the NHS 111 service in 2021 and were triaged to a primary care disposition.

Primary and secondary outcome measures

Face validity of conceptual model. Comparison between real and simulated data for quarterly counts (and 95% confidence intervals) for patient contact with emergency ambulance (999), 111, and primary and secondary care services. Mean difference and 95% confidence intervals in healthcare system usage between simulations and difference in mean proportion of avoidable admissions for callers who presented to an emergency department (ED).

Results

The simulation of the current system estimated that there would be 39,283 (95%CI 39,237–39,328) primary care contacts, 2,042 (95%CI 2,032–2,051) 999 calls and 1,120 (95%CI 1,114–1,127) avoidable ED attendances. Modifying the model to ensure a timely primary care response resulted in a mean increase in primary care contacts of 37,748

For peer review only

(95%CI 37,667–37,829), a mean reduction in 999 calls of -449 (95%CI -461– -436) and a mean reduction in avoidable ED attendance of -26 (95%CI -35– -17).

Conclusion

In this simulated study, ensuring timely contact with a primary care service would lead to a significant reduction in 999 and 111 calls, and ED attendances (although not avoidable ED attendance). However, this is likely to be impractical given the need to almost double current primary care service provision. Further economic and qualitative research is needed to determine whether this intervention would be cost effective and acceptable to both patients and primary care clinicians.

STRENGTHS AND LIMITATIONS OF THIS STUDY

- This study benefits from utilisation of a robust method (discrete event simulation) that has been widely used and accepted as a method to simulate healthcare system changes
- The model was informed by relevant stakeholders and a linked dataset.
- The model and its source code has been made available free and open source for others to interrogate and develop
- It is a simple working model, and does not take account of factors that have previously been associated with inappropriate ED attendance, such as age, sex, ethnicity and clinical input into the 111 call
- The data that informed the model only covers a discrete region in England, which may affect generalisability.

INTRODUCTION

The National Health Service (NHS) 111 service aims to assist members of the public with urgent medical care needs and is the successor to the NHS Direct service in England. Following pilots in four sites it was rolled out nationally, with the final site going live in England in 2014, and in 2019/20 received over 19 million calls (1). Its key founding

For peer review only - http://bmjopen.bmj.com/site/about/guidelines.xhtml

objective was to provide easy access to support for the public with urgent care needs, to ensure they received the “right care, from the right person, in the right place, at the right time” (2). It is also the key component of the 24/7 Integrated Urgent Care Service outlined in the NHS Long Term Plan (3).

Approximately 55% of all calls made to the English National Health Service (NHS) 111 call service are triaged to a referral to a primary care service. If a timely service cannot be provided to patients, it is possible that this will result in patients calling 999 or attending emergency departments (ED) directly. We have previously reported that just under half (47.6%) of callers to 111 triaged to a primary care service disposition made contact with a primary care service as their first healthcare interaction (4). There was evidence that patients more frequently attended an ED when they had not had previous contact with a primary care service. However, rates of avoidable attendance were similar whether callers had contact with a primary care service prior to attendance at ED or not.

Other studies examining caller behaviour and the impacts on the wider healthcare system (5,6), have utilised datasets from 2017 and earlier, and patterns of activity identified then, may not be applicable now.

This study aimed to develop a Discrete Event Simulation to model the current healthcare system for callers triaged to a primary care disposition. Discrete Event Simulation models allow for the simulation of queuing problems, in which entities (such as patients) queue for processes that require resources (7). Our primary objective was to develop a model which represented the current healthcare system in relation to callers to 111 who received a primary care service disposition following triage. Our secondary objective was to use the model to estimate the predicted impact and associated resourcing required to ensure a timely primary care contact for all callers to the 111 service in a region of Yorkshire.

For peer review only - http://bmjopen.bmj.com/site/about/guidelines.xhtml

METHODS

Conceptual modelling

Prior to developing a simulation that represents the trajectories of patients within the model, it is necessary to develop a conceptual model: an appropriately simplified collection of assumptions about the components and structure of the 'real-world' (8) that can act as a blue print for the in-silico model development. This technique has its origins in soft systems methodology (9), and enabled us to work with stakeholders in the ambulance service, NHS 111, general practice and emergency departments to co-produce processes and capture their knowledge of the health and social care system (Figure 1). This co-production is not only crucial in terms of improving the accuracy of the model, but also an important component of stakeholder engagement and the formation of strategic partnerships (10).

Data

In tandem with the conceptual modelling phase, we obtained routine, retrospective data from the Connected Yorkshire research database, which provides linked data for approximately 1.2 million citizens across the Bradford and Airedale region of Yorkshire. Datasets include 111 and emergency ambulance (999) call data, as well as primary and secondary care (including emergency department and in-patient activity).

We identified all 111 calls between the 1st January 2021 and 31st December 2021 for patients who were triaged to a primary care disposition and registered with a General Practitioner (GP) in the Bradford area at the time of the call. Subsequent healthcare system access in the following 72 hours following the first (index) call was identified by searching datasets containing details of 111 and 999 calls, and primary care, hospital emergency department and in-patient admissions (4).

For peer review only - http://bmjopen.bmj.com/site/about/guidelines.xhtml

Discrete event simulation

Using Brennan et al's (11) taxonomy, we determined that Discrete Event Simulation (DES) was a good choice of operational research methodology to address the research question. DES is a flexible modelling technique that can represent complex behaviour of callers to 111, and their interactions with the healthcare system simultaneously. DES can also incorporate capacity and resource constraints and account for chance, for example the stochastic behaviour of callers (7,12,13). DES is also useful, because it makes it possible to ask 'what if' questions such as the research question. It has been used widely in healthcare, examining health care systems operations, and disease progression, screening and health behaviour modelling (14,15).

We created an *in-silico* version of the conceptual model using the programming language Python (v3.9.6) and the SymPy library (v4.0.1, 16). In addition, we created a web-based dashboard in order to present the results of the simulation and provide an opportunity for users/stakeholders to run their own simulations. To achieve this, we used Plotly Dash (v5.11.0, 17) and created a fully contained environment in docker (v4.2.0.7078) which is available from the study GitHub repository (https://github.com/RichardPilbery/MOOOD-study).

Patients enter the model at the point they are triaged to a primary care disposition by 111 and have exited the service. The 'arrival' rate of callers into the model is determined by sampling from an exponential probability distribution with lambda set to the 1/mean arrival rate for 111 calls, stratified by hour, yearly quarter and whether the call occurred at a weekend. When a patient is created in the simulation, they are assigned a primary care disposition with an associated triage acuity (Figure 2). This consists of a time frame within which a contact with a primary care service should be made, and specifies whether the contact should be face-to-face or remotely, for example by telephone. The allocation is random, but weighted according to the proportions seen in the Connected Yorkshire data. Once the patient has been allocated a primary care disposition, the uniform distribution is sampled to determine whether the primary care contact will be achieved in the specified

For peer review only - http://bmjopen.bmj.com/site/about/guidelines.xhtml

triage time frame. This is assumed to be the case when the sampled value is less than proportion calculated from the Connected Yorkshire dataset.

Patient trajectory through the healthcare system is determined using transition probabilities calculated from the Connected Yorkshire dataset using the statistics software, R (18). The next health care service (including no further contact) is determined by random sampling, weighted by whether a primary care service contact was made immediately after the index 111 call, and the yearly quarter. If the next healthcare service access is determined to be the emergency department (ED), then the uniform distribution is sampled to determine whether the admission will be avoidable. As before, this is assumed to be the case when the sampled value is less than proportion calculated from the Connected Yorkshire dataset. The proportions are stratified by primary care disposition and whether initial contact was made with a primary care service.

For this study, an ED attendance is classed as being 'avoidable' when it meets the (19) criteria. They defined an avoidable attendance as a patient presenting to a consultant-led ED which provides a 24 hour service with full resuscitation facilities and designated accommodation for the reception of emergency care patients (referred to as a type 1 ED (20)), but who do not receive investigations, treatments or referral that required the facilities of a type 1 ED.

Unless the patient is allocated to 'no further healthcare contact', in which case the patient exits the simulation, the 'queue' time i.e. the time the patient waits until the service is accessed and the time that the healthcare activity takes, is calculated. The 'queue' time is calculated by sampling from a distribution specific to the current healthcare service being access and the next, for example index 111 call to emergency department attendance. Activity times are calculated by sampling from distributions specific to the service being accessed. In both cases, the optimal distribution was chosen by the Python library fitter (v1.5.2, 21) based on the distribution with the lowest sum of square errors.

Patients remain in the simulation until either they are allocated to 'no further healthcare contact' or the elapsed time exceeds 72 hours. The model runs for a period of 1 year of simulated time.

For peer review only - http://bmjopen.bmj.com/site/about/guidelines.xhtml

Analysis

Once the model was developed, we performed 100 runs of 1 year's worth of simulated 111 calls and monitored the patient's trajectory over the subsequent 72 hours. As the model is stochastic, multiple runs of the model were necessary to ensure that results are representative and not due to stochastic noise. A summary of all simulations was reported as the mean and 95% confidence intervals for counts and proportions.

Since all models are approximations of the 'real world', we used the historic data provided by the Connected Yorkshire dataset to see how closely our model matches with real data (the primary objective), visually and descriptively analysing the difference in temporal distribution of calls between the actual and simulated data, as well as comparing quarterly aggregated healthcare service access by patients, following the index 111 call. Finally, we performed a visual assessment of patient trajectory, to determine whether it approximated actual patient behaviour.

For the secondary objective, we undertook the 'what if' analysis, examining the hypothetical situation whereby all index 111 calls with a primary care disposition received a timely (i.e. within the specified call triage time) response from a primary care service. No capacity constraints were placed on primary care services in order to estimate the true resourcing that would be required to meet the demand appropriately. We calculated the mean difference and 95% confidence intervals in healthcare system usage between simulations and determined the difference in mean proportion of avoidable admissions for callers who presented to an ED.

Ethical approval

This study was approved by the Bradford Learning Health System Board in accordance with the Connected Yorkshire NHS Research Ethics Committee (REC) and Confidentiality Advisory Group (CAG) approvals relating to the Connected Yorkshire research database (17/EM/0254). No separate Health Research Authority (HRA) approval was required for this study.

For peer review only - http://bmjopen.bmj.com/site/about/guidelines.xhtml

PPI

The application and protocol for this study was review by the Yorkshire Ambulance Service NHS Trust patient research ambassador. In addition, Connected Bradford have an active patient and public involvement group and a representative attended the approvals board for this study.

RESULTS

111 call activity in both simulations was similar to the actual call activity seen in the real data, although overall, the simulation did slightly overestimate the number of calls, particularly between 00:00 and 06:00 (Supplementary 1).

Healthcare system access

Overall, the simulation tended to underestimate 999 call activity and overestimate all other healthcare service activity. Primary care, emergency department admission and subsequent 111 call activity was most closely simulated (Table 1, Supplementary 2).

Table 1: Comparison of simulation activity with Connected Yorkshire data

Healthcare service	cYorkshire2021 (N)	Simulation (N, 95%CI)
Ambulance service	2,623	2,042 (2,032–2,051)
Emergency department	9,290	9,337 (9,319–9,355)
Primary care	38,680	39,283 (39,237–39,328)
In-patient	4,367	4,668 (4,654–4,681)
111	4,710	4,789 (4,771–4,806)

For peer review only - http://bmjopen.bmj.com/site/about/guidelines.xhtml

"What if?" scenario

Assuming no constraints on primary care service ability, we simulated a hypothetical scenario whereby all 111 calls with a primary care disposition received a timely primary care contact (Table 2).

Table 2: Comparison between simulation and what if scenario

Service	Simulation (mean, 95%CI)	What if (mean, 95%CI)	Mean difference (mean 95%CI)	Mean proportion change (mean %, 95%CI)
Ambulance service	2,042 (2,032–2,051)	1,593 (1,584–1,602)	-449 (-461– -436)	-22 (-30.2– -13.8)
Emergency department	9,337 (9,319–9,355)	8,228 (8,213–8,244)	-1,109 (-1,133– -1,085)	-11.9 (-18.3– -5.5)
Primary care	39,283 (39,237–39,328)	77,030 (76,964–77,097)	37,748 (37,667–37,829)	96.1 (92.2– 99.9)
In-patient	4,668 (4,654–4,681)	4,620 (4,607--4,632)	-48 (-66– -30)	-1 (-3–1)
111	4,789 (4,771–4,806)	3,074 (3,061–3,087)	-1,715 (-1,737– -1,693)	-35.8 (-45.3– -26.3)

ED attendance

In the original Connected Yorkshire dataset, there were 9,290 emergency department attendances and 1,029 (11.1%) met the (19) definition of an avoidable attendance. The simulated dataset estimated a mean ED attendance of 9,337 cases (95%CI 9,319–9,356), of which, 1,120 (95%CI 1,114–1,127; mean proportion, 12%, 95%CI 5.6–18.4%) were classed

For peer review only - http://bmjopen.bmj.com/site/about/guidelines.xhtml
For peer review only

as avoidable. In the 'what if' scenario, there was a reduction of more than 10% in ED attendances, but little change in the proportion of avoidable attendance (Table 3).

Table 3: Simulated ED attendance rates stratified by avoidable admission

Model	Avoidable attendance (mean, 95%CI)	Total attendances (mean, 95%CI)	Proportion of avoidable attendances (mean, 95%CI)
Simulation	1,120 (1,114–1,127)	9,337 (9,319–9,356)	12% (5.6–18.4%)
What if	1,094 (1,087–1,100)	8,228 (8,213–8,244)	13.3% (6.6–ß20%)

Discussion

Using a simple simulation model, we have been able to successfully capture the healthcare system as it relates to callers to 111 who are triaged to a primary care disposition. We used the model to estimate the impact of a hypothetical scenario in which primary care services had sufficient capacity to provide a service to all 111 callers triaged to their service and that all callers elected to make contact with the service. We determined that the 'base case' model sufficiently closely replicated the real world data to be confident that its estimates of the impact of the scenario tested would be useful.

In the simulated 'what if?' scenario, we found that ensuring a timely primary care contact for all 111 callers triaged to a primary care disposition, would lead to a significant reduction in ambulance service and 111 contacts and emergency department admissions. However, to achieve this it would be necessary to almost double the number of primary care contacts provided by the current healthcare system which, in the wider context of clinical staff shortages in general practice (22,23) and the NHS (24), is unlikely to be feasible. While we did not conduct an economic analysis, the data suggests that the savings made by reducing certain healthcare service contacts are unlikely to offset the additional cost in providing additional primary care services.

Even if the capacity issues could be overcome, adjusting caller behaviour could be even more challenging. Almost 39% of callers in the base case model did not seek any further healthcare contact in the 72 hours after the index 111 call. Previous qualitative work on

For peer review only - http://bmjopen.bmj.com/site/about/guidelines.xhtml

understanding why patients call an ambulance for 'primary care problems' highlighted the perceived limitations of out-of-hours primary care services by patients to address their needs and, in the case of patients with chronic conditions, previous negative experiences of community-based healthcare services being unable to 'help' would need to be addressed (25). In addition, there are reasons why patients may not follow advice to contact primary care which are beyond the control of the health system, such as work or family/childcare commitments, transport issues (including weather-related) and demographic factors such as younger age, female sex and low socio-economic background (26); factors that would be unaffected by changes in service provision. That said, there is limited evidence that increasing availability of primary care services (such as offering extended hours) can lead to a reduction in access other services. This is particularly pertinent, since most 111 calls with a primary care disposition occur out-of-hours (27).

This work has generated stakeholder discussion about whether the apparent supply/demand problem identified by the simulation could be addressed by utilising the Additional Roles Reimbursement Scheme. In addition, they have also suggested that further qualitative work might help to understand which primary care roles best suit particular caller presentations (for example a consultation with physiotherapist rather than a GP for a musculoskeletal presentation), which may reduce the proportion of callers who do not access healthcare services following the 111 call and are potentially left with an unmet healthcare need.

Strengths and weaknesses

This study benefits from utilisation of a robust method (discrete event simulation) that has been widely used and accepted as a method to simulate healthcare system changes (7). In addition, the model was informed by relevant stakeholders and a linked dataset. However, it is nevertheless a simple working model, and does not take account of factors that have previously been associated with inappropriate ED attendance, such as age, sex, ethnicity and clinical input into the 111 call (6).

The model may benefit from more extensive validation and verification than the simple 'black box' validation described here, in which outputs from the model were compared

For peer review only - http://bmjopen.bmj.com/site/about/guidelines.xhtml

with the equivalent real world metrics. Additional validation measures, such as performing sensitivity analyses to determine the impact of inaccuracies in modelling assumptions could be useful in increasing confidence in the model's predictions (28). In addition, verification measures such as extensive review of code by others can help to identify issues in the software engineering side of the development (29). However, the model and its source code has been made available free and open source for others to interrogate and develop, and the simple validation steps we have taken give us relative confidence in our estimates of the scale of the resourcing required to meet demand.

The data that informed the model only covers a discrete region in West Yorkshire, which may affect generalisability. Bradford is mainly an urban area and the 13th most deprived local authority in England (out of 333) based on the Index of Multiple Deprivation (30). In addition, it is based on data collected in 2021, during the pandemic, which may not be representative of the current healthcare trajectory for patients who call 111. However, the open source nature of the model means that others can use the model to parameterise according to the data from their own regions.

Further economic and qualitative analysis would be beneficial to determine whether an intervention such as this would be cost-effective, feasible and acceptable to patients.

CONCLUSION

In this simulated study, ensuring timely contact with a primary care service is estimated to lead to a significant reduction in 999 and 111 calls, and ED attendances (although not avoidable ED attendance). However, this is likely to be impractical given the estimated need to almost double current primary care service provision. Further economic and qualitative research is needed to determine whether this intervention would be cost effective and acceptable to both patients and primary care providers.

Acknowledgements

This work uses data provided by patients and collected by the NHS as part of their care and support. The authors would also like to thank Dr. Eithne Cummins and Dr. Jon Dickson for

For peer review only

providing a stakeholder perspective to the study, and Dr. Hazel Squires for early support in operational research methodology. In addition, we are grateful for the support provided by the team at Connected Yorkshire, especially Kuldeep Sohal and John Birkinshaw.

The work was supported as a project of the NIHR Applied Research Collaboration for the South West Peninsula Health Service Modelling Associates (HSMA) Programme (https://sites.google.com/nihr.ac.uk/hsma) which also provided the training required in Python and Discrete Event Simulation.

Contributors

RP and DC conceived and designed the study. RP obtained the research approvals to access the datasets and acts as guarantor for the paper. RP and MS developed the code necessary to extract the data that informed the model. RP coded the model. DC provided mentoring support and guidance for the project, and delivered the training in Python and SimPy. CO assisted with the design of the study. All authors drafted the manuscript and contributed substantially to its revision.

Funding

This paper presents independent research by the NIHR Applied Research Collaboration Yorkshire and Humber (ARC YH). This work was supported by the National Institute for Health Research Applied Research Collaboration South West Peninsula and Yorkshire and Humber.

Disclaimer

The views expressed in this publication are those of the author(s) and not necessarily those of the National Institute for Health Research or the Department of Health and Social Care.

Data sharing

The complete model, including transition probabilities and inter-arrival, activity and queue distributions are available from the study GitHub repository

For peer review only

For peer review only - http://bmjopen.bmj.com/site/about/guidelines.xhtml

(https://github.com/RichardPilbery/MOOOD-study). The software is supplied under a GNU General Public License.

REFERENCES

1. NHS England. NHS 111 Minimum Data Set 2019-20 [Internet]. 2020 [cited 2020 Dec 29]. Available from: https://www.england.nhs.uk/statistics/statistical-work-areas/nhs-111-minimum-data-set/nhs-111-minimum-data-set-2019-20/

2. UK Government. NHS 111 [Internet]. 2011 [cited 2020 Dec 10]. Available from: https://web.archive.org/web/20130415084921/http://www.connectingforhealth.nhs.uk/systemsandservices/pathways/news/nhs111intro.pdf

3. England N. The NHS Long Term Plan [Internet]. NHS Long Term Plan. 2019 [cited 2020 Dec 10]. Available from: https://www.longtermplan.nhs.uk/publication/nhs-long-term-plan/

4. Pilbery R, Smith M, Green J, Chalk D. An analysis of NHS 111 demand for primary care services [Internet]. medRxiv; 2023 [cited 2023 Apr 5]. Available from: https://www.medrxiv.org/content/10.1101/2023.03.20.23287449v1

5. Lewis J, Stone T, Simpson R, Jacques R, O'Keeffe C, Croft S, et al. Patient compliance with NHS 111 advice: Analysis of adult call and ED attendance data 2013–2017. PLOS ONE [Internet]. 2021 May [cited 2021 May 28];16(5):e0251362. Available from: https://journals.plos.org/plosone/article?id=10.1371/journal.pone.0251362

6. Nakubulwa MA, Greenfield G, Pizzo E, Magusin A, Maconochie I, Blair M, et al. To what extent do callers follow the advice given by a non-emergency medical helpline (NHS 111): A retrospective cohort study. Montefiori M, editor. PLOS ONE [Internet]. 2022 Apr [cited 2023 Mar 2];17(4):e0267052. Available from: https://dx.plos.org/10.1371/journal.pone.0267052

7. Caro JJ, Möller J, Karnon J, Stahl J, Ishak J. Discrete event simulation for health technology assessment. Boca Raton ; London: CRC Press, Taylor & Francis Group; 2016.

For peer review only - http://bmjopen.bmj.com/site/about/guidelines.xhtml

8. Banks J, Carson J, Nelson B, Nicol D. Discrete-event system simulation. 5th ed. Harlow: Pearson; 2014. (Always learning).

9. Checkland P. Systems Thinking, Systems Practice: Includes a 30 Year Retrospective. New Ed edition. Chichester ; New York: JW; 1999.

10. Green D. How Change Happens. Reprint edition. Oxford, United Kingdom: OUP Oxford; 2018.

11. Brennan A, Chick SE, Davies R. A taxonomy of model structures for economic evaluation of health technologies. Health Economics [Internet]. 2006 Dec [cited 2019 Mar 7];15(12):1295–310. Available from: http://doi.wiley.com/10.1002/hec.1148

12. Pitt M, Monks T, Crowe S, Vasilakis C. Systems modelling and simulation in health service design, delivery and decision making. BMJ Quality & Safety [Internet]. 2016 Jan [cited 2018 Nov 21];25(1):38–45. Available from: http://qualitysafety.bmj.com/lookup/doi/10.1136/bmjqs-2015-004430

13. Karnon J, Stahl J, Brennan A, Caro JJ, Mar J, Möller J. Modeling Using Discrete Event Simulation: A Report of the ISPOR-SMDM Modeling Good Research Practices Task Force–4. Medical Decision Making [Internet]. 2012 Sep [cited 2018 Nov 18];32(5):701–11. Available from: https://doi.org/10.1177/0272989X12455462

14. Forbus JJ, Berleant D. Discrete-Event Simulation in Healthcare Settings: A Review. Modelling [Internet]. 2022 Dec [cited 2023 Mar 8];3(4):417–33. Available from: https://www.mdpi.com/2673-3951/3/4/27

15. Vázquez-Serrano JI, Peimbert-García RE, Cárdenas-Barrón LE. Discrete-Event Simulation Modeling in Healthcare: A Comprehensive Review. International Journal of Environmental Research and Public Health [Internet]. 2021 Nov [cited 2023 Mar 8];18(22):12262. Available from: https://www.ncbi.nlm.nih.gov/pmc/articles/PMC8625660/

For peer review only - http://bmjopen.bmj.com/site/about/guidelines.xhtml

16. Meurer A, Smith CP, Paprocki M, Čertík O, Kirpichev SB, Rocklin M, et al. SymPy: Symbolic computing in Python. PeerJ Computer Science [Internet]. 2017 Jan;3:e103. Available from: https://doi.org/10.7717/peerj-cs.103

17. Plotly Technologies Inc. Collaborative data science [Internet]. 2015 [cited 2023 Mar 17]. Available from: https://plot.ly

18. R Core Team. R: A language and environment for statistical computing [Internet]. Vienna, Austria; 2022. Available from: https://www.R-project.org/

19. O'Keeffe C, Mason S, Jacques R, Nicholl J. Characterising non-urgent users of the emergency department (ED): A retrospective analysis of routine ED data. PLOS ONE [Internet]. 2018 Feb [cited 2022 Jun 24];13(2):e0192855. Available from: https://journals.plos.org/plosone/article?id=10.1371/journal.pone.0192855

20. NHS Digital. Emergency care department type [Internet]. 2023 [cited 2023 Mar 14]. Available from: https://www.datadictionary.nhs.uk/attributes/emergency_care_department_type.html

21. Cokelaer T, Kravchenko A, Lahdjirayhan, Msat59, Varma A, L B, et al. Cokelaer/fitter: v1.5.2 [Internet]. Zenodo; 2023 [cited 2023 Mar 3]. Available from: https://zenodo.org/record/7497983

22. The Health Foundation. A quarter of GP and general practice nursing posts could be vacant in 10 years [Internet]. 2022 [cited 2023 Mar 13]. Available from: https://www.health.org.uk/news-and-comment/news/a-quarter-of-gp-and-general-practice-nursing-posts-could-be-vacant-in-10-years

23. British Medical Association. Pressures in general practice data analysis [Internet]. The British Medical Association is the trade union and professional body for doctors in the UK. 2023 [cited 2023 Mar 13]. Available from: https://www.bma.org.uk/advice-and-support/nhs-delivery-and-workforce/pressures/pressures-in-general-practice-data-analysis

For peer review only - http://bmjopen.bmj.com/site/about/guidelines.xhtml

24. Waitzman E. Staff shortages in the NHS and social care sectors [Internet]. 2022 [cited 2023 Mar 13]. Available from: https://lordslibrary.parliament.uk/staff-shortages-in-the-nhs-and-social-care-sectors/

25. Booker MJ, Simmonds RL, Purdy S. Patients who call emergency ambulances for primary care problems: A qualitative study of the decision-making process. Emergency Medicine Journal [Internet]. 2014 Jun [cited 2023 Mar 18];31(6):448–52. Available from: https://emj.bmj.com/content/31/6/448

26. Parsons J, Bryce C, Atherton H. Which patients miss appointments with general practice and the reasons why: A systematic review. British Journal of General Practice [Internet]. 2021 Jun [cited 2023 Jan 13];71(707):e406–12. Available from: https://bjgp.org/content/71/707/e406

27. Whittaker W, Anselmi L, Kristensen SR, Lau YS, Bailey S, Bower P, et al. Associations between Extending Access to Primary Care and Emergency Department Visits: A Difference-In-Differences Analysis. PLOS Medicine [Internet]. 2016 Sep [cited 2023 Mar 10];13(9):e1002113. Available from: https://journals.plos.org/plosmedicine/article?id=10.1371/journal.pmed.1002113

28. Macdonald I, Strachan P. Practical application of uncertainty analysis. Energy and Buildings [Internet]. 2001 Feb [cited 2023 Apr 12];33(3):219–27. Available from: https://www.sciencedirect.com/science/article/pii/S0378778800000852

29. Raunak M, Olsen M. Quantifying validation of discrete event simulation models. In: Proceedings of the Winter Simulation Conference 2014. 2014. p. 628–39.

30. City of Bradford Metropolitan District Council. Indices of Deprivation 2019 - Intelligence bulletin [Internet]. 2019 [cited 2023 Mar 15]. Available from: https://ubd.bradford.gov.uk/media/1534/indices-of-deprivation-2019-intelligence-bulletin.pdf

For peer review only

For peer review only - http://bmjopen.bmj.com/site/about/guidelines.xhtml

FIGURE LEGENDS

- Figure 1: Design-orientated conceptual model of patients with an urgent care problem
- Figure 2: Programmatic simulation flow chart

For peer review only

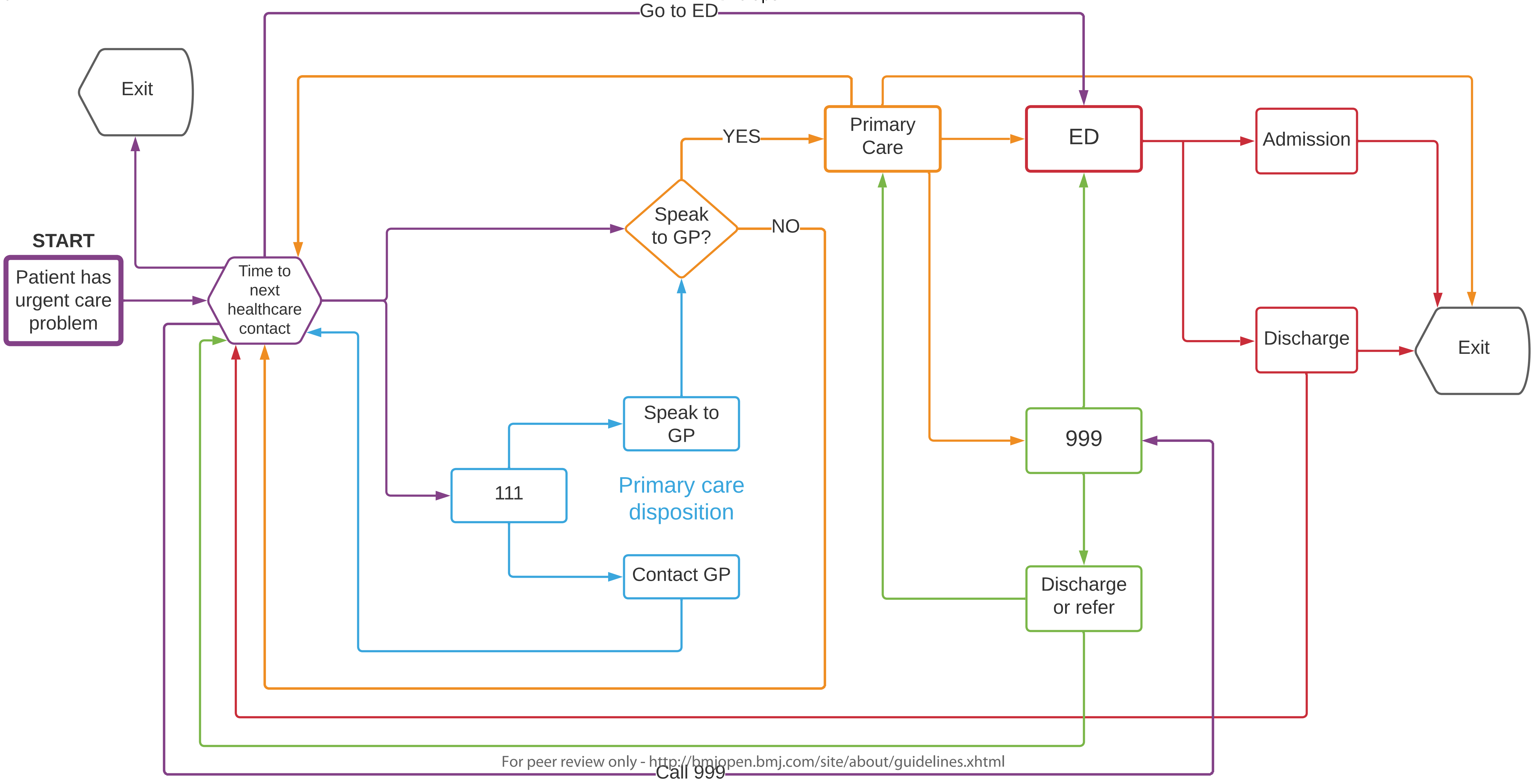
Go to ED
Exit
START
Patient has urgent care problem
Time to next healthcare contact
Speak to GP?
YES
NO
Primary Care
ED
Admission
Discharge
Exit
Speak to GP
111
Primary care disposition
Contact GP
999
Discharge or refer
Call 999

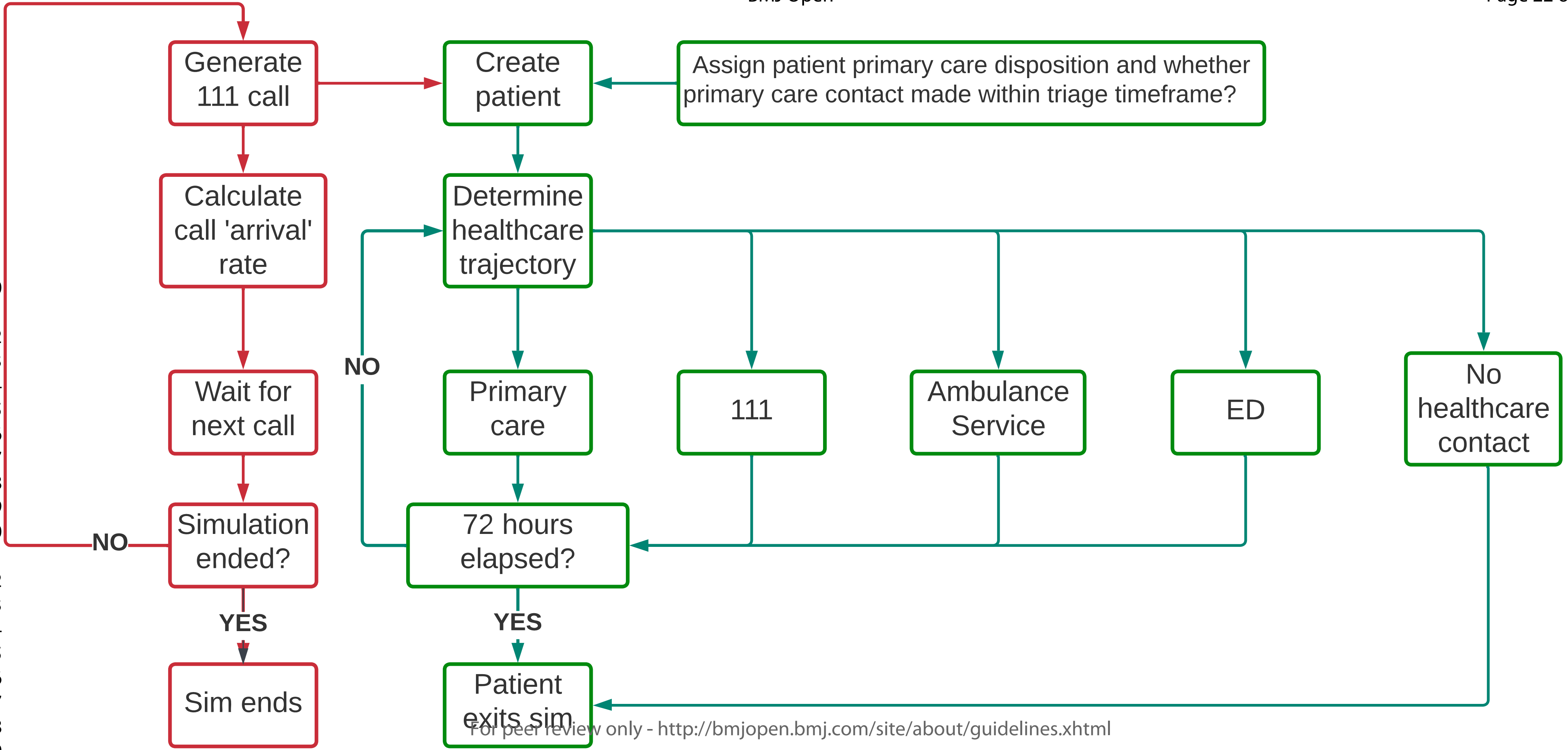
Generate 111 call
Create patient
Assign patient primary care disposition and whether primary care contact made within triage timeframe?
Calculate call 'arrival' rate
Determine healthcare trajectory
NO
Wait for next call
Primary care
111
Ambulance Service
ED
No healthcare contact
Simulation ended?
NO
72 hours elapsed?
YES
YES
Sim ends
Patient exits sim

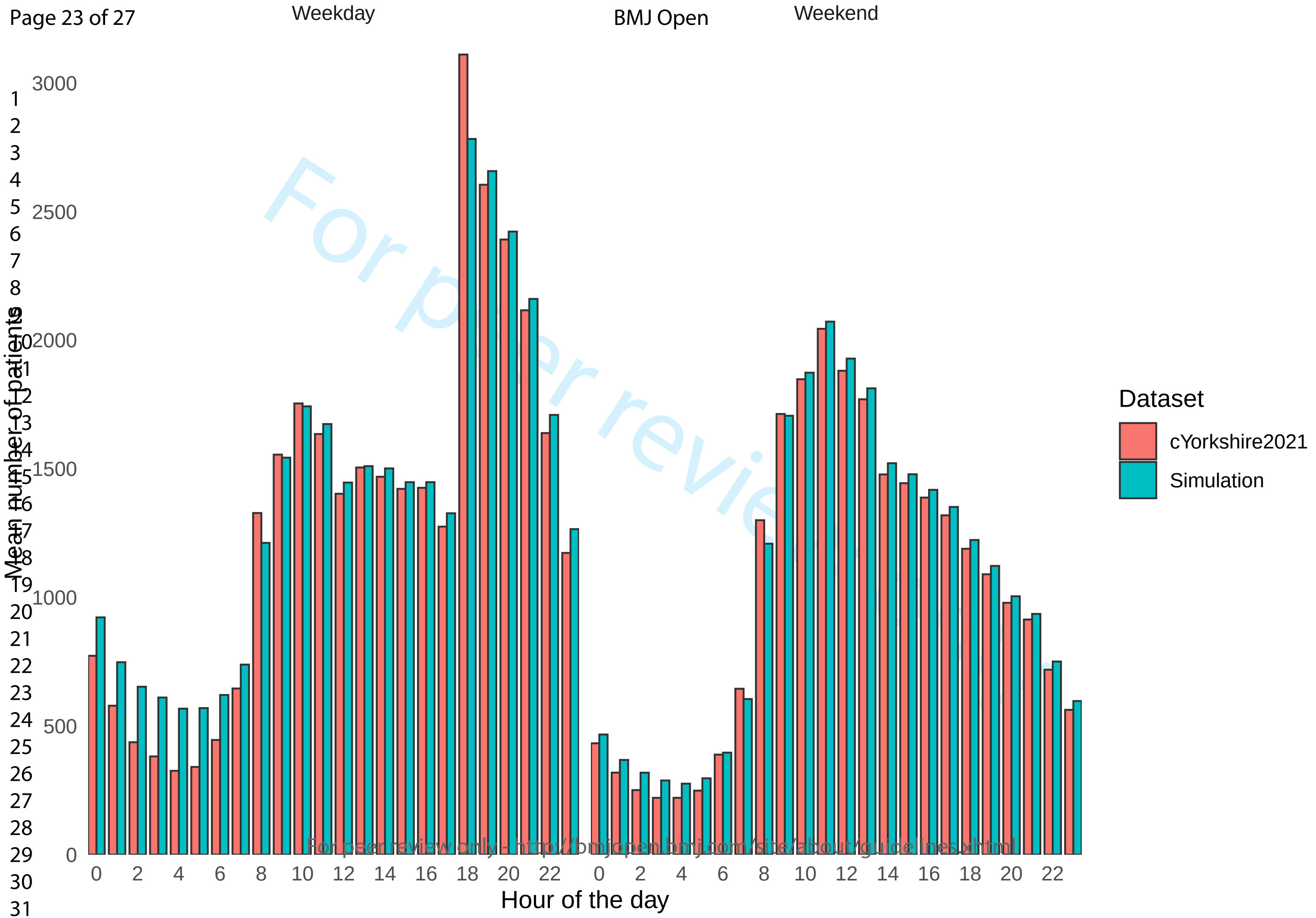

For peer review only - http://bmjopen.bmj.com/site/about/guidelines.xhtml

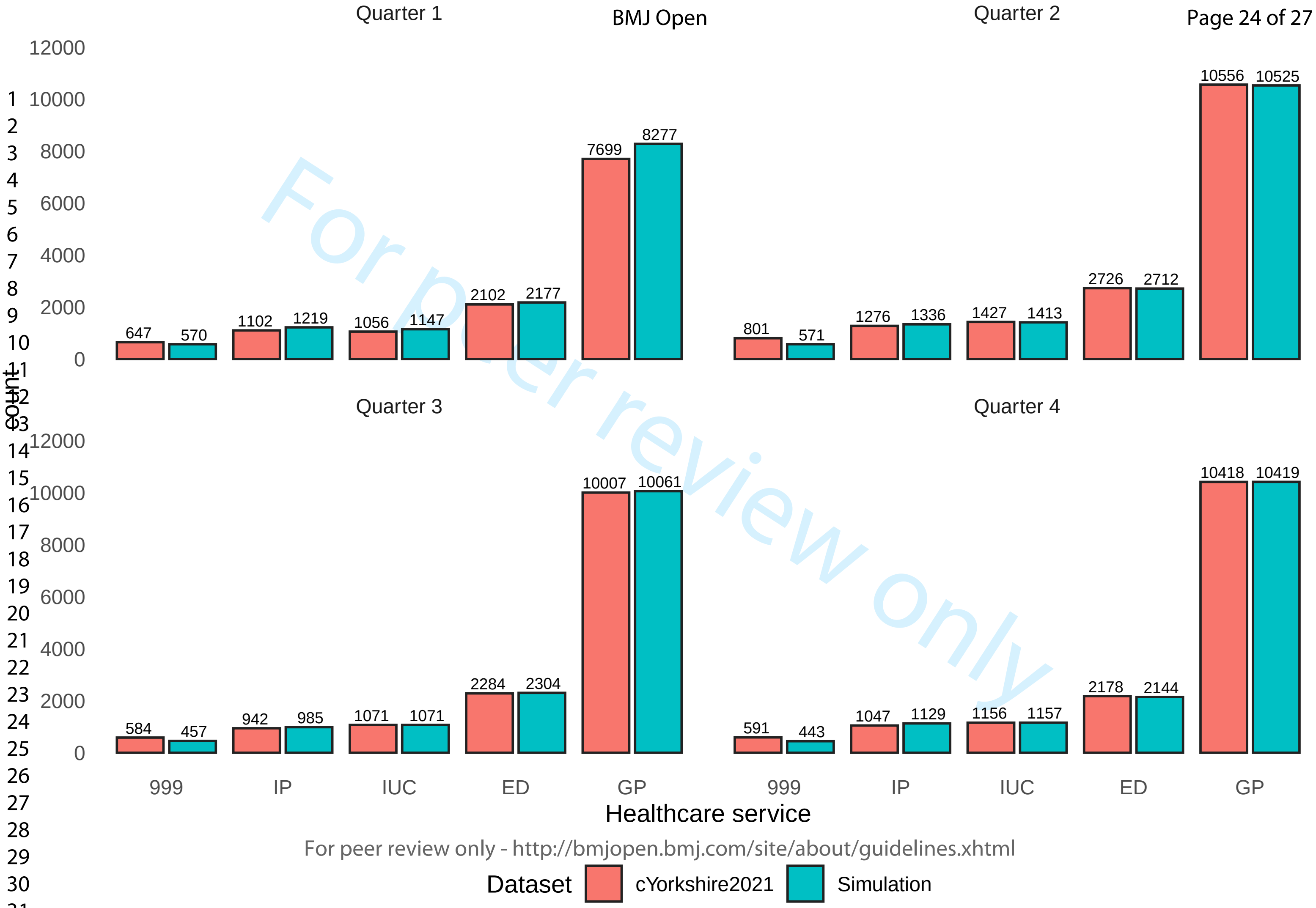

For peer review only - http://bmjopen.bmj.com/site/about/guidelines.xhtml

STROBE extension for simulation based research checklist

Item	Item no.	STROBE item	Location	Extension for SBR	Location
Title and abstract	1	a. Indicate the study's design with a commonly used term in the title or the abstract.<br>b. Provide in the abstract an informative and balanced summary of what was done and what was found.	a. Title<br><br>b. Abstract P2	In abstract or key terms, the MESH or searchable keyword term must have the word simulation or simulated.	Keywords: P1
Introduction					
Background/rationale	2	Explain the scientific background and rationale for the investigation being reported.	P2	Clarify whether simulation is subject of research or investigational method for research.	P2
Objectives	3	State specific objectives, including any prespecified hypotheses.	P2		
Methods					
Study design	4	Present key elements of study design early in the paper.	Methods		
Setting	5	Describe the setting, locations, and relevant dates, including periods of recruitment, exposure, follow-up, and data collection.	Data section		
Participants	6	a. Cohort study: give the eligibility criteria and the sources and methods of selection of participants. Describe methods of follow-up.<br>b. Cohort study: for matched studies, give matching criteria and number of exposed and unexposed.	a. Data section<br><br>b. N/A		
Variables	7	Clearly define all outcomes, exposures, predictors, potential confounders, and effect modifiers.<br>Give diagnostic criteria, if applicable.		Describe the theoretical and/or conceptual rationale for the design of the intervention/exposure.<br>Describe the intervention/exposure with sufficient detail to permit	Methods section: Conceptual modelling DES

STROBE extension for simulation based research checklist

				replication.<br>Clearly describe all simulation-specific exposures, potential confounders, and effect modifiers.	
Data sources/ measurement	8	For each variable of interest, give sources of data and details of methods of assessment (measurement).<br>Describe comparability of assessment methods if there is >1 group.	Data section	In describing the details of methods of assessment, include (when applicable) the setting, instrument, simulator type, timing in relation to the intervention, along with any methods used to enhance the quality of measurements.<br>Provide evidence to support the validity and reliability of assessment tools in this context (if available).	DES Analysis
Bias	9	Describe any efforts to address potential sources of bias.	Strengths and weaknesses		
Study size	10	Explain how the study size was arrived.			
Quantitative variables	11	Explain how quantitative variables were handled in the analyses. If applicable, describe which groupings were chosen and why.			
Statistical methods	12	a. Describe all statistical methods, including those used to control for confounding.<br>b. Describe any methods used to examine subgroups and interactions.<br>c. Explain how missing data were addressed.<br>d. Cohort study: if applicable, explain how loss to follow-up was addressed.<br>Case–control study: if applicable, explain how matching of cases and controls was addressed.<br>Cross-sectional study: if applicable, describe analytical methods taking	a. Methods<br>b. Methods<br>c. N/A<br>d. N/A	Clearly indicate the unit of analysis (e.g., individual, team, system), identify repeated measures on subjects, and describe how these issues were addressed.	

STROBE extension for simulation based research checklist

		account of sampling strategy.<br>e. Describe any sensitivity analyses.	e. N/A		
Results					
Participants	13	a. Report the numbers of individuals at each stage of the study (e.g., numbers potentially eligible, examined for eligibility, confirmed eligible, included in the study, completing follow-up, and analyzed).<br>b. Give reasons for nonparticipation at each stage.<br>c. Consider use of a flow diagram.	Sankey diagram (kind of).		
Descriptive data	14	a. Give characteristics of study participants (e.g., demographic, clinical, social) and information on exposures and potential confounders.<br>b. Indicate the number of participants with missing data for each variable of interest.<br>c. Cohort study: summarize follow-up time (e.g., average and total amount).	Results	In describing characteristics of study participants, include their previous experience with simulation and other relevant features as related to the intervention(s).	N/A
Outcome data	15	Cohort study: report numbers of outcome events or summary measures over time.<br>Case–control study: report numbers in each exposure category or summary measures of exposure.<br>Cross-sectional study: report numbers of outcome events or summary measures.	Results		
Main results	16	a. Give unadjusted estimates and, if applicable, confounder-adjusted estimates and their precision (e.g., 95 % confidence intervals).<br>Make clear which	A. Table 1, 2, 3	For assessments involving >1 rater, interrater reliability should be reported.	N/A

STROBE extension for simulation based research checklist

		confounders were adjusted for and why they were included.<br>b. Report category boundaries when continuous variables were categorized.<br>c. If relevant, consider translating estimates of relative risk into absolute risk for a meaningful time period.	b. N/A<br>c. N/A		
Other analyses	17	Report other analyses done (e.g., analyses of subgroups and interactions and sensitivity analyses).	Results		
Discussion					
Key results	18	Summarize key results with reference to study objectives.	First 2 paragraphs of Discussion		
Limitations	19	Discuss limitations of the study, taking into account sources of potential bias or imprecision. Discuss both direction and magnitude of any potential bias.	Strengths and weakness	Specifically discuss the limitations of SBR.	Strengths and weakness
Interpretation	20	Give a cautious overall interpretation of results considering objectives, limitations, multiplicity of analyses, results from similar studies, and other relevant evidence.	Discussion		
Generalisability	21	Discuss the generalisability (external validity) of the study results.	Strengths and weaknesses	Describe generalisability of simulation-based outcomes to patient-based outcomes (if applicable).	Strengths and weaknesses
Other information					
Funding	22	Give the source of funding and the role of the funders for the present study and, if applicable, for the original study on which the present article is based.	Funding	List simulator brand and if conflict of interest for intellectual property exists.	Methods – DES

Modelling NHS 111 demand for primary care services: a discrete event simulation

Journal:	*BMJ Open*
Manuscript ID	bmjopen-2023-076203.R1
Article Type:	Original research
Date Submitted by the Author:	03-Aug-2023
Complete List of Authors:	Pilbery, Richard; Yorkshire Ambulance Service NHS Trust, Research and Development<br>Smith, Madeleine; NHS Devon<br>Green, Jonathan; University of Plymouth Faculty of Health<br>Chalk, Daniel; University of Exeter Medical School,<br>O'Keeffe, Colin; The University of Sheffield, School of Health and Related Research
**Primary Subject Heading**:	Health informatics
Secondary Subject Heading:	Health services research
Keywords:	Primary Care < Primary Health Care, Telemedicine < BIOTECHNOLOGY & BIOINFORMATICS, Health informatics < BIOTECHNOLOGY & BIOINFORMATICS

SCHOLARONE™
Manuscripts

I, the Submitting Author has the right to grant and does grant on behalf of all authors of the Work (as defined in the below author licence), an exclusive licence and/or a non-exclusive licence for contributions from authors who are: i) UK Crown employees; ii) where BMJ has agreed a CC-BY licence shall apply, and/or iii) in accordance with the terms applicable for US Federal Government officers or employees acting as part of their official duties; on a worldwide, perpetual, irrevocable, royalty-free basis to BMJ Publishing Group Ltd ("BMJ") its licensees and where the relevant Journal is co-owned by BMJ to the co-owners of the Journal, to publish the Work in this journal and any other BMJ products and to exploit all rights, as set out in our licence.

The Submitting Author accepts and understands that any supply made under these terms is made by BMJ to the Submitting Author unless you are acting as an employee on behalf of your employer or a postgraduate student of an affiliated institution which is paying any applicable article publishing charge ("APC") for Open Access articles. Where the Submitting Author wishes to make the Work available on an Open Access basis (and intends to pay the relevant APC), the terms of reuse of such Open Access shall be governed by a Creative Commons licence – details of these licences and which Creative Commons licence will apply to this Work are set out in our licence referred to above.

Other than as permitted in any relevant BMJ Author's Self Archiving Policies, I confirm this Work has not been accepted for publication elsewhere, is not being considered for publication elsewhere and does not duplicate material already published. I confirm all authors consent to publication of this Work and authorise the granting of this licence.

For peer review only

Modelling NHS 111 demand for primary care services: a discrete event simulation

Author information

Richard Pilbery (corresponding author)
Research paramedic
Yorkshire Ambulance Service NHS Trust
Springhill, Brindley Way
Wakefield 41 Business Park
Wakefield
WF2 0XQ
ORCID: https://orcid.org/0000-0002-5797-9788
email: r.pilbery@nhs.net

Madeleine Smith
NHS Devon

Jonathan Green
University of Plymouth
ORCID: https://orcid.org/0000-0001-5738-7515

Dr. Daniel Chalk
University of Exeter
ORCID: https://orcid.org/0000-0002-4165-4364

Colin O'Keeffe
University of Sheffield
ORCID: https://orcid.org/0000-0002-4484-7401

Keywords: 111, Telephone triage, primary care, emergency care, discrete event simulation

For peer review only

ABSTRACT

Objectives

This feasibility study aimed to model *in-silico* the current healthcare system for patients triaged to a primary care disposition following a call to NHS 111, and determine the effect of reconfiguring the healthcare system to ensure a timely primary care service contact.

Design

Discrete event simulation.

Setting

Single English NHS 111 call centre in Yorkshire.

Participants

Callers registered with a Bradford general practitioner (GP) who contacted the NHS 111 service in 2021 and were triaged to a primary care disposition.

Primary and secondary outcome measures

Face validity of conceptual model. Comparison between real and simulated data for quarterly counts (and 95% confidence intervals) for patient contact with emergency ambulance (999), 111, and primary and secondary care services. Mean difference and 95% confidence intervals in healthcare system usage between simulations and difference in mean proportion of avoidable admissions for callers who presented to an emergency department (ED).

Results

The simulation of the current system estimated that there would be 39,283 (95%CI 39,237–39,328) primary care contacts, 2,042 (95%CI 2,032–2,051) 999 calls and 1,120 (95%CI 1,114–1,127) avoidable ED attendances. Modifying the model to ensure a timely primary care response resulted in a mean percentage increase of 196.1% (95% CI 192.2–

For peer review only

199.9%) in primary care contacts, and a mean percentage decrease of 78.0% (95% CI 69.8–86.2%) in 999 calls and 88.1% (95% CI 81.7–94.5%) in ED attendances. Avoidable ED attendances reduced by a mean of -26 (95%CI -35– -17).

Conclusion

In this simulated study, ensuring timely contact with a primary care service would lead to a significant reduction in 999 and 111 calls, and ED attendances (although not avoidable ED attendance). However, this is likely to be impractical given the need to almost double current primary care service provision. Further economic and qualitative research is needed to determine whether this intervention would be cost effective and acceptable to both patients and primary care clinicians.

STRENGTHS AND LIMITATIONS OF THIS STUDY

- This study benefits from utilisation of a robust method (discrete event simulation) that has been widely used and accepted as a method to simulate healthcare system changes
- The model was informed by relevant stakeholders and a linked dataset.
- The model and its source code has been made available free and open source for others to interrogate and develop
- It is a simple working model, and does not take account of factors that have previously been associated with inappropriate ED attendance, such as age, sex, ethnicity and clinical input into the 111 call
- The data that informed the model only covers a discrete region in England, which may affect generalisability.

INTRODUCTION

The National Health Service (NHS) 111 service aims to assist members of the public with urgent medical care needs and is the successor to the NHS Direct service in England. Following pilots in four sites it was rolled out nationally, with the final site going live in

For peer review only - http://bmjopen.bmj.com/site/about/guidelines.xhtml

England in 2014, and in 2019/20 received over 19 million calls (1). Its key founding objective was to provide easy access to support for the public with urgent care needs, to ensure they received the “right care, from the right person, in the right place, at the right time” (2). It is also the key component of the 24/7 Integrated Urgent Care Service outlined in the NHS Long Term Plan (3).

The triage outcome of approximately 55% of calls made to the English National Health Service (NHS) 111 call service, is a referral to a primary care service. The triage process includes the method of contact (for example, callers may be asked to contact a primary care service themselves, or a referral made by 111 to a primary care service to contact the patient directly), and a timeframe within which this should occur. If a timely service (defined as a primary care contact within the time specified as part of the triage outcome) cannot be provided to patients, it is possible that this will result in patients calling 999 or attending emergency departments (ED) directly. We have previously reported that just under half (47.6%) of callers to 111 triaged to a primary care service disposition made contact with a primary care service as their first healthcare interaction (4). There was evidence that patients more frequently attended an ED when they had not had previous contact with a primary care service. However, rates of avoidable attendance were similar whether callers had contact with a primary care service prior to attendance at ED or not.

Other studies examining caller behaviour and the impacts on the wider healthcare system (5,6), have utilised datasets from 2017 and earlier, and patterns of activity identified then, may not be applicable now.

This study aimed to develop a Discrete Event Simulation to model the current healthcare system for callers triaged to a primary care disposition. Discrete Event Simulation models allow for the simulation of queuing problems, in which entities (such as patients) queue for processes that require resources (7). Our primary objective was to develop a model which represented the current healthcare system in relation to callers to 111 who received a primary care service disposition following triage. Our secondary objective was to use the model to estimate the predicted impact and associated resourcing required to ensure a timely primary care contact for all callers to the 111 service in a region of Yorkshire.

For peer review only - http://bmjopen.bmj.com/site/about/guidelines.xhtml

METHODS

Conceptual modelling

Prior to developing a simulation that represents the trajectories of patients within the model, it is necessary to develop a conceptual model: an appropriately simplified collection of assumptions about the components and structure of the 'real-world' (8) that can act as a blue print for the in-silico model development. This technique has its origins in soft systems methodology (9), and enabled us to work with stakeholders in the ambulance service, NHS 111, general practice and emergency departments to co-produce processes and capture their knowledge of the health and social care system (Figure 1). This co-production is not only crucial in terms of improving the accuracy of the model, but also an important component of stakeholder engagement and the formation of strategic partnerships (10).

Data

In tandem with the conceptual modelling phase, we obtained routine, retrospective data from the Connected Yorkshire research database, which provides linked patient-level data for approximately 1.2 million citizens across the Bradford and Airedale region of Yorkshire. Datasets include 111 and emergency ambulance (999) call data, as well as primary and secondary care (including emergency department and in-patient activity). Data submitted to Connected Yorkshire contain a unique identifier in the form of a pseudonymised NHS number (used throughout the NHS as a unique identifier for patient care). In this study, we used it, along with the date and time of access to services, to track a caller's healthcare journey.

We identified all 111 calls between the 1st January 2021 and 31st December 2021 for patients who were triaged to a primary care disposition and registered with a General Practitioner (family physician, GP) in the Bradford area at the time of the call. Subsequent healthcare system access in the 72 hours following the first (index) call, was identified by

For peer review only - http://bmjopen.bmj.com/site/about/guidelines.xhtml

programmatically searching for the patient's unique identifier in the 111, 999 calls, primary care, hospital emergency department and in-patient admission datasets (4).

Triage to a primary care service in this study refers to a range of services, including referral to the patient's own GP, or an out-of-hours GP service, pharmacy, community, optician, mental health or maternity service. Depending on the triage disposition and service availability, referrals are either made directly by 111 with a request for a service to contact the caller, or the caller is advised to contact/attend the service directly. In the source data, over 96% of primary care disposition referrals, were to a GP (or out-of-hours service) and of the other services, only pharmacies, mental health and optician service interactions would not appear in the data. However, these services only comprised 2% of triage outcomes in the source data (4).

Discrete event simulation

Using (11) taxonomy, we determined that Discrete Event Simulation (DES) was a good choice of operational research methodology to address the research question. DES is a flexible modelling technique that can represent complex behaviour of callers to 111, and their interactions with the healthcare system simultaneously. DES can also incorporate capacity and resource constraints and account for chance, for example the stochastic behaviour of callers (7,7,12). DES is also useful, because it makes it possible to ask 'what if' questions such as the research question. It has been used widely in healthcare, examining health care systems operations, and disease progression, screening and health behaviour modelling (7,13).

We created an *in-silico* version of the conceptual model using the programming language Python (v3.9.6) and the SymPy library (v4.0.1, 14). In addition, we created a web-based dashboard in order to present the results of the simulation and provide an opportunity for users/stakeholders to run their own simulations. To achieve this, we used Plotly Dash (v5.11.0, 15) and created a fully contained environment in docker (v4.2.0.7078) which is available from the study GitHub repository (https://github.com/RichardPilbery/MOOOD-study).

For peer review only - http://bmjopen.bmj.com/site/about/guidelines.xhtml

Patients enter the model at the point they are triaged to a primary care disposition by 111 and have exited the service. The 'arrival' rate of callers into the model is determined by sampling from an exponential probability distribution with lambda set to the 1/mean arrival rate for 111 calls, stratified by hour, yearly quarter and whether the call occurred at a weekend. When a patient is created in the simulation, they are assigned a primary care disposition with an associated triage acuity (Figure 2). This consists of a time frame within which a contact with a primary care service should be made, and specifies whether the contact should be face-to-face or remotely, for example by telephone. The allocation is random, but weighted according to the proportions seen in the Connected Yorkshire data. Once the patient has been allocated a primary care disposition, the uniform distribution is sampled to determine whether the primary care contact will be achieved in the specified triage time frame. This is assumed to be the case when the sampled value is less than proportion calculated from the Connected Yorkshire dataset.

Patient trajectory through the healthcare system is determined using transition probabilities calculated from the Connected Yorkshire dataset using the statistics software, R (16). The next health care service (including no further contact) is determined by random sampling, weighted by whether a primary care service contact was made immediately after the index 111 call, and the yearly quarter. If the next healthcare service access is determined to be the emergency department (ED), then the uniform distribution is sampled to determine whether the admission will be avoidable. As before, this is assumed to be the case when the sampled value is less than proportion calculated from the Connected Yorkshire dataset. The proportions are stratified by primary care disposition and whether initial contact was made with a primary care service.

For this study, an ED attendance is classed as being 'avoidable' when it meets the O'Keeffe et al (17) criteria. They defined an avoidable attendance as a patient presenting to a consultant-led ED which provides a 24 hour service with full resuscitation facilities and designated accommodation for the reception of emergency care patients (referred to as a type 1 ED (18)), but who do not receive investigations, treatments or referral that required the facilities of a type 1 ED.

For peer review only - http://bmjopen.bmj.com/site/about/guidelines.xhtml

Unless the patient is allocated to 'no further healthcare contact', in which case the patient exits the simulation, the 'queue' time i.e. the time the patient waits until the service is accessed and the time that the healthcare activity takes, is calculated. The 'queue' time is calculated by sampling from a distribution specific to the current healthcare service being access and the next, for example index 111 call to ED attendance. Activity times are calculated by sampling from distributions specific to the service being accessed. In both cases, the optimal distribution was chosen by the Python library fitter (v1.5.2, 19) based on the distribution with the lowest sum of square errors.

Patients remain in the simulation for a maximum of 72 hours unless they are allocated to 'no further healthcare contact' prior to this. The model generates 111 calls for a period of 1 year of simulated time.

Analysis

Once the model was developed, we performed 100 runs of 1 year's worth of simulated 111 calls and monitored the patient's trajectory over the subsequent 72 hours. As the model is stochastic, multiple runs of the model were necessary to ensure that results are representative and not due to stochastic noise. A summary of all simulations was reported as the mean and 95% confidence intervals for counts and proportions.

Since all models are approximations of the 'real world', we used the historic data provided by the Connected Yorkshire dataset to see how closely our model matches with real data (the primary objective), visually and descriptively analysing the difference in temporal distribution of calls between the actual and simulated data, as well as comparing quarterly aggregated healthcare service access by patients, following the index 111 call. Finally, we performed a visual assessment of patient trajectory, to determine whether it approximated actual patient behaviour.

For the secondary objective, we undertook the 'what if' analysis, examining the hypothetical situation whereby all index 111 calls with a primary care disposition received a timely (i.e. within the specified call triage time) response from a primary care service. No capacity constraints were placed on primary care services in order to estimate the true

For peer review only - http://bmjopen.bmj.com/site/about/guidelines.xhtml

resourcing that would be required to meet the demand appropriately. We calculated the mean difference and 95% confidence intervals in healthcare system usage between simulations and determined the difference in mean proportion of avoidable admissions for callers who presented to an ED.

Ethical approval

This study was approved by the Bradford Learning Health System Board in accordance with the Connected Yorkshire NHS Research Ethics Committee (REC) and Confidentiality Advisory Group (CAG) approvals relating to the Connected Yorkshire research database (17/EM/0254). No separate Health Research Authority (HRA) approval was required for this study.

Patient and Public Involvement

The application and protocol for this study was review by the Yorkshire Ambulance Service NHS Trust patient research ambassador. In addition, Connected Bradford have an active patient and public involvement group and a representative attended the approvals board for this study.

RESULTS

We visually compared outputs from the model with historic patient data and observed sufficient similarity in the distribution pattern of calls across the day, which gave us confidence that the model was sufficiently capturing the variability within the real-world data (Figure 3, Supplementary 1 and 2). 111 call activity in both simulations was similar to the actual call activity seen in the real data, although overall, the simulation did slightly overestimate the number of calls, particularly between 00:00 and 06:00 (Figure 3a). The simulation tended to underestimate 999 call activity and overestimate all other healthcare service activity. Primary care, ED admission and subsequent 111 call activity was most closely simulated (Table 1). Primary care, ED attendance and subsequent 111 call activity was most closely simulated (Figure 3b, Supplementary 1).

For peer review only - http://bmjopen.bmj.com/site/about/guidelines.xhtml

Table 1: Comparison of total healthcare service contacts between the origin simulation and 'what if?' scenario

Service	Connected Yorkshire data (N)	Simulation (mean, 95%CI)	What if? (mean, 95%CI)	Mean difference (mean 95%CI)	Mean percentage change (mean %, 95%CI)
Ambulance service	2,623	2,042 (2,032–2,051)	1,593 (1,584–1,602)	-449 (-461– -436)	78 (69.8–86.2)
Emergency department	9,290	9,337 (9,319–9,355)	8,228 (8,213–8,244)	-1,109 (-1,133– -1,085)	88.1 (81.7–94.5)
Primary care	38,680	39,283 (39,237–39,328)	77,030 (76,964–77,097)	37,748 (37,667–37,829)	196.1 (192.2–199.9)
In-patient	4,367	4,668 (4,654–4,681)	4,620 (4,607–4,632)	-48 (-66– -30)	99 (97-- 101)
111	4,710	4,789 (4,771–4,806)	3,074 (3,061–3,087)	-1,715 (-1,737– -1,693)	64.2 (54.7–ß 73.7)

The first healthcare contact following the index 111 call in the Connected Yorkshire data was most commonly a primary care service (26,690/56102; 47.6%). However, a large proportion (21,749/56,102; 38.8%) had no healthcare contact in the 72 hours following the index 111 call. This was mirrored by the simulation data, where the first contact following the index 111 call was a primary care service in a mean of 27,697/58,302

For peer review only - http://bmjopen.bmj.com/site/about/guidelines.xhtml

(47.5%) of cases, with no further healthcare contact the second most common finding (22,700/58,302; 38.9%) (Supplementary 2).

"What if?" scenario

We simulated a hypothetical scenario whereby all 111 calls with a primary care disposition received a timely primary care contact (the 'what if?' scenario). This assumed that there were no capacity constraints on the service and that all callers accessed a primary care service following the index 111 call.

This resulted in an increase in primary care contacts from a mean of 39,283 (95%CI 39,237–39,328) in the base simulation to 77,030 (95%CI 76,964–77,097); a mean percentage increase of 196.1% (95%CI, 192.2– 199.9%) (Table 1). All other healthcare services saw a reduction in healthcare interactions, with the most notable reductions occurring in 999 and 111 service access (mean percentage change 78.0% and 64.2%, respectively).

ED attendance

In the original Connected Yorkshire dataset, there were 9,290 emergency department attendances and 1,029 (11.1%) met the O'Keefe et al (17) definition of an avoidable attendance. The simulated dataset estimated a mean ED attendance of 9,337 cases (95%CI 9,319–9,356), of which, 1,120 (95%CI 1,114–1,127; mean proportion, 12%, 95%CI 5.6–18.4%) were classed as avoidable. In the 'what if' scenario, ED attendances reduced to a mean of 8,228 (95%CI 8,213–8,244) attendances but there was little change in the proportion of avoidable attendance (mean proportion 13.3%, 95%CI 6.6–20%).

Discussion

Using a simple simulation model, we have been able to successfully capture the healthcare system as it relates to callers to 111 who are triaged to a primary care disposition. We used the model to estimate the impact of a hypothetical scenario in which primary care services had sufficient capacity to provide a service to all 111 callers triaged to their service and

For peer review only

that all callers elected to contact the service. We determined that the 'base case' model sufficiently closely replicated the real-world data to be confident that its estimates of the impact of the scenario tested would be useful. While we did not monitor or simulate ambulance dispatch, we can still see the outcome of any physical attendance by an ambulance crew by monitoring ED attendances or GP contacts following a 999 call, for example.

In the simulated 'what if?' scenario, we found that ensuring a timely primary care contact for all 111 callers triaged to a primary care disposition, would lead to a significant reduction in ambulance service and 111 contacts and emergency department admissions. However, to achieve this it would be necessary to almost double the number of primary care contacts provided by the current healthcare system which, in the wider context of clinical staff shortages in general practice (20,21) and the NHS (22), is unlikely to be feasible. While we did not conduct an economic analysis, the data suggests that the savings made by reducing certain healthcare service contacts are unlikely to offset the additional cost in providing additional primary care services.

Even if the capacity issues could be overcome, adjusting caller behaviour could be even more challenging. Almost 39% of callers in the base case model did not seek any further healthcare contact in the 72 hours after the index 111 call. Previous qualitative work on understanding why patients call an ambulance for 'primary care problems' highlighted the perceived limitations of out-of-hours primary care services by patients to address their needs and, in the case of patients with chronic conditions, previous negative experiences of community-based healthcare services being unable to 'help' would need to be addressed (23). In addition, there are reasons why patients may not follow advice to contact primary care which are beyond the control of the health system, such as work or family/childcare commitments, transport issues (including weather-related) and demographic factors such as younger age, female sex and low socio-economic background (24); factors that would be unaffected by changes in service provision. That said, there is limited evidence that increasing availability of primary care services (such as offering extended hours) can lead to a reduction in access other services. This is particularly pertinent, since most 111 calls with a primary care disposition occur out-of-hours (25).

For peer review only - http://bmjopen.bmj.com/site/about/guidelines.xhtml

This work has generated stakeholder discussion about whether the apparent supply/demand problem identified by the simulation could be addressed by utilising the NHS Additional Roles Reimbursement Scheme which provides funding for healthcare professionals (HCP) to work as a multi-disciplinary team within primary care to expand capacity and support delivery of new services tailored to the local population. Potential HCP roles include pharmacists, paramedics, occupational therapists, and dieticians in addition to others.

In addition, they have also suggested that further qualitative work might help to understand which primary care roles best suit particular caller presentations (for example a consultation with physiotherapist rather than a GP for a musculoskeletal presentation), which may reduce the proportion of callers who do not access healthcare services following the 111 call and are potentially left with an unmet healthcare need.

Strengths and weaknesses

This study benefits from utilisation of a robust method (discrete event simulation) that has been widely used and accepted as a method to simulate healthcare system changes (26). In addition, the model was informed by relevant stakeholders and a linked dataset. However, it is nevertheless a simple working model, and does not take account of factors that have previously been associated with inappropriate ED attendance, such as age, sex, ethnicity and clinical input into the 111 call (6).

The model may benefit from more extensive validation and verification than the simple 'black box' validation described here, in which outputs from the model were compared with the equivalent real world metrics. Additional validation measures, such as performing sensitivity analyses to determine the impact of inaccuracies in modelling assumptions could be useful in increasing confidence in the model's predictions (27). In addition, verification measures such as extensive review of code by others can help to identify issues in the software engineering side of the development (28). In addition, only callers who are registered with a Bradford GP were included. Given that GP registrations are consistently higher than Office for National Statistics (ONS) population estimates, it is difficult to determine what proportion of the population are not registered with a GP. Patients also

For peer review only - http://bmjopen.bmj.com/site/about/guidelines.xhtml

have a right to opt-out of their clinical data being used for research purposes, and this comprises around 4% of patients registered with a GP in England (29).

The data that informed the model only covers a discrete region in West Yorkshire, which may affect generalisability. Bradford is mainly an urban area and the 13th most deprived local authority in England (out of 333) based on the Index of Multiple Deprivation (30). In addition, it is based on data collected in 2021, during the pandemic, which may not be representative of the current healthcare trajectory for patients who call 111. However, the open source nature of the model means that others can use the model to parameterise according to the data from their own regions.

Further economic and qualitative analysis would be beneficial to determine whether an intervention such as this would be cost-effective, feasible and acceptable to patients. This could potentially include ambulance attendance following 999 calls, which may have implications for ambulance service operational performance and cost effectiveness.

CONCLUSION

In this simulated study, ensuring timely contact with a primary care service is estimated to lead to a significant reduction in 999 and 111 calls, and ED attendances (although not avoidable ED attendance). However, this is likely to be impractical given the estimated need to almost double current primary care service provision. Further economic and qualitative research is needed to determine whether this intervention would be cost effective and acceptable to both patients and primary care providers.

Acknowledgements

This work uses data provided by patients and collected by the NHS as part of their care and support. The authors would also like to thank Dr. Eithne Cummins and Dr. Jon Dickson for providing a stakeholder perspective to the study, and Dr. Hazel Squires for early support in operational research methodology. In addition, we are grateful for the support provided by the team at Connected Yorkshire, especially Kuldeep Sohal and John Birkinshaw.

For peer review only - http://bmjopen.bmj.com/site/about/guidelines.xhtml

The work was supported as a project of the NIHR Applied Research Collaboration for the South West Peninsula Health Service Modelling Associates (HSMA) Programme (https://sites.google.com/nihr.ac.uk/hsma) which also provided the training required in Python and Discrete Event Simulation.

Contributors

RP and DC conceived and designed the study. JG conducted a literature review. RP obtained the research approvals to access the datasets and acts as guarantor for the paper. RP and MS developed the code necessary to extract the data that informed the model. RP coded the model. DC provided mentoring support and guidance for the project, and delivered the training in Python and SimPy. CO assisted with the design of the study. All authors drafted the manuscript and contributed substantially to its revision.

Funding

This paper presents independent research by the NIHR Applied Research Collaboration Yorkshire and Humber (ARC YH). This work was supported by the National Institute for Health Research Applied Research Collaboration South West Peninsula and Yorkshire and Humber.

Disclaimer

The views expressed in this publication are those of the author(s) and not necessarily those of the National Institute for Health Research or the Department of Health and Social Care.

Data sharing

The complete model, including transition probabilities and inter-arrival, activity and queue distributions are available from the study GitHub repository (https://github.com/RichardPilbery/MOOOD-study). The software is supplied under a GNU General Public License.

For peer review only

Competing interests

The authors declare not completing interests with respect to this work.

REFERENCES

1. NHS England. NHS 111 Minimum Data Set 2019-20 [Internet]. 2020 [cited 2020 Dec 29]. Available from: https://www.england.nhs.uk/statistics/statistical-work-areas/nhs-111-minimum-data-set/nhs-111-minimum-data-set-2019-20/

2. UK Government. NHS 111 [Internet]. 2011 [cited 2020 Dec 10]. Available from: https://web.archive.org/web/20130415084921/http://www.connectingforhealth.nhs.uk/systemsandservices/pathways/news/nhs111intro.pdf

3. England N. The NHS Long Term Plan [Internet]. NHS Long Term Plan. 2019 [cited 2020 Dec 10]. Available from: https://www.longtermplan.nhs.uk/publication/nhs-long-term-plan/

4. Pilbery R, Smith M, Green J, Chalk D. An analysis of NHS 111 demand for primary care services [Internet]. medRxiv; 2023 [cited 2023 Apr 5]. Available from: https://www.medrxiv.org/content/10.1101/2023.03.20.23287449v1

5. Lewis J, Stone T, Simpson R, Jacques R, O'Keeffe C, Croft S, et al. Patient compliance with NHS 111 advice: Analysis of adult call and ED attendance data 2013–2017. PLOS ONE [Internet]. 2021 May [cited 2021 May 28];16(5):e0251362. Available from: https://journals.plos.org/plosone/article?id=10.1371/journal.pone.0251362

6. Nakubulwa MA, Greenfield G, Pizzo E, Magusin A, Maconochie I, Blair M, et al. To what extent do callers follow the advice given by a non-emergency medical helpline (NHS 111): A retrospective cohort study. Montefiori M, editor. PLOS ONE [Internet]. 2022 Apr [cited 2023 Mar 2];17(4):e0267052. Available from: https://dx.plos.org/10.1371/journal.pone.0267052

7. Vázquez-Serrano JI, Peimbert-García RE, Cárdenas-Barrón LE. Discrete-Event Simulation Modeling in Healthcare: A Comprehensive Review. International Journal of

For peer review only

Environmental Research and Public Health [Internet]. 2021 Nov [cited 2023 Mar 8];18(22):12262. Available from: https://www.ncbi.nlm.nih.gov/pmc/articles/PMC8625660/

8. Kotiadis K, Tako AA, Vasilakis C. A participative and facilitative conceptual modelling framework for discrete event simulation studies in healthcare. Journal of the Operational Research Society [Internet]. 2014 Feb [cited 2023 Jul 27];65(2):197–213. Available from: https://doi.org/10.1057/jors.2012.176

9. Kotiadis K. Using soft systems methodology to determine the simulation study objectives. Journal of Simulation [Internet]. 2007 Aug [cited 2023 Jul 27];1(3):215–22. Available from: https://doi.org/10.1057/palgrave.jos.4250025

10. Franco LA, Montibeller G. Facilitated modelling in operational research. European Journal of Operational Research [Internet]. 2010 Sep [cited 2023 Jul 27];205(3):489–500. Available from: https://www.sciencedirect.com/science/article/pii/S0377221709006699

11. Brennan A, Chick SE, Davies R. A taxonomy of model structures for economic evaluation of health technologies. Health Economics [Internet]. 2006 Dec [cited 2019 Mar 7];15(12):1295–310. Available from: http://doi.wiley.com/10.1002/hec.1148

12. Karnon J, Stahl J, Brennan A, Caro JJ, Mar J, Möller J. Modeling Using Discrete Event Simulation: A Report of the ISPOR-SMDM Modeling Good Research Practices Task Force–4. Medical Decision Making [Internet]. 2012 Sep [cited 2018 Nov 18];32(5):701–11. Available from: https://doi.org/10.1177/0272989X12455462

13. Forbus JJ, Berleant D. Discrete-Event Simulation in Healthcare Settings: A Review. Modelling [Internet]. 2022 Dec [cited 2023 Mar 8];3(4):417–33. Available from: https://www.mdpi.com/2673-3951/3/4/27

14. Meurer A, Smith CP, Paprocki M, Čertík O, Kirpichev SB, Rocklin M, et al. SymPy: Symbolic computing in Python. PeerJ Computer Science [Internet]. 2017 Jan;3:e103. Available from: https://doi.org/10.7717/peerj-cs.103

For peer review only

15. Plotly Technologies Inc. Collaborative data science [Internet]. 2015 [cited 2023 Mar 17]. Available from: https://plot.ly

16. R Core Team. R: A language and environment for statistical computing [Internet]. Vienna, Austria; 2022. Available from: https://www.R-project.org/

17. O'Keeffe C, Mason S, Jacques R, Nicholl J. Characterising non-urgent users of the emergency department (ED): A retrospective analysis of routine ED data. PLOS ONE [Internet]. 2018 Feb [cited 2022 Jun 24];13(2):e0192855. Available from: https://journals.plos.org/plosone/article?id=10.1371/journal.pone.0192855

18. NHS Digital. Emergency care department type [Internet]. 2023 [cited 2023 Mar 14]. Available from: https://www.datadictionary.nhs.uk/attributes/emergency_care_department_type.html

19. Cokelaer T, Kravchenko A, Lahdjirayhan, Msat59, Varma A, L B, et al. Cokelaer/fitter: v1.5.2 [Internet]. Zenodo; 2023 [cited 2023 Mar 3]. Available from: https://zenodo.org/record/7497983

20. The Health Foundation. A quarter of GP and general practice nursing posts could be vacant in 10 years [Internet]. 2022 [cited 2023 Mar 13]. Available from: https://www.health.org.uk/news-and-comment/news/a-quarter-of-gp-and-general-practice-nursing-posts-could-be-vacant-in-10-years

21. British Medical Association. Pressures in general practice data analysis [Internet]. The British Medical Association is the trade union and professional body for doctors in the UK. 2023 [cited 2023 Mar 13]. Available from: https://www.bma.org.uk/advice-and-support/nhs-delivery-and-workforce/pressures/pressures-in-general-practice-data-analysis

22. Waitzman E. Staff shortages in the NHS and social care sectors [Internet]. 2022 [cited 2023 Mar 13]. Available from: https://lordslibrary.parliament.uk/staff-shortages-in-the-nhs-and-social-care-sectors/

For peer review only - http://bmjopen.bmj.com/site/about/guidelines.xhtml

23. Booker MJ, Simmonds RL, Purdy S. Patients who call emergency ambulances for primary care problems: A qualitative study of the decision-making process. Emergency Medicine Journal [Internet]. 2014 Jun [cited 2023 Mar 18];31(6):448–52. Available from: https://emj.bmj.com/content/31/6/448

24. Parsons J, Bryce C, Atherton H. Which patients miss appointments with general practice and the reasons why: A systematic review. British Journal of General Practice [Internet]. 2021 Jun [cited 2023 Jan 13];71(707):e406–12. Available from: https://bjgp.org/content/71/707/e406

25. Whittaker W, Anselmi L, Kristensen SR, Lau YS, Bailey S, Bower P, et al. Associations between Extending Access to Primary Care and Emergency Department Visits: A Difference-In-Differences Analysis. PLOS Medicine [Internet]. 2016 Sep [cited 2023 Mar 10];13(9):e1002113. Available from: https://journals.plos.org/plosmedicine/article?id=10.1371/journal.pmed.1002113

26. Caro JJ, Möller J, Karnon J, Stahl J, Ishak J. Discrete event simulation for health technology assessment. Boca Raton ; London: CRC Press, Taylor & Francis Group; 2016.

27. Macdonald I, Strachan P. Practical application of uncertainty analysis. Energy and Buildings [Internet]. 2001 Feb [cited 2023 Apr 12];33(3):219–27. Available from: https://www.sciencedirect.com/science/article/pii/S0378778800000852

28. Raunak M, Olsen M. Quantifying validation of discrete event simulation models. In: Proceedings of the Winter Simulation Conference 2014. 2014. p. 628–39.

29. NHS Digital. [MI] National Data Opt-out, May 2023 [Internet]. NDRS. 2023 [cited 2023 Jul 19]. Available from: https://digital.nhs.uk/data-and-information/publications/statistical/national-data-opt-out/may-23

30. City of Bradford Metropolitan District Council. Indices of Deprivation 2019 - Intelligence bulletin [Internet]. 2019 [cited 2023 Mar 15]. Available from: https://ubd.bradford.gov.uk/media/1534/indices-of-deprivation-2019-intelligence-bulletin.pdf

For peer review only - http://bmjopen.bmj.com/site/about/guidelines.xhtml

FIGURE LEGENDS

- Figure 1: Design-orientated conceptual model of patients with an urgent care problem
- Figure 2: Programmatic simulation flow chart
- Figure 3: a) Comparison between Connected Yorkshire and simulation data of hourly caller numbers stratified by weekday/weekend. b) Comparison between Connected Yorkshire and simulation data of healthcare service contacts, stratified by service and yearly quarter.

For peer review only

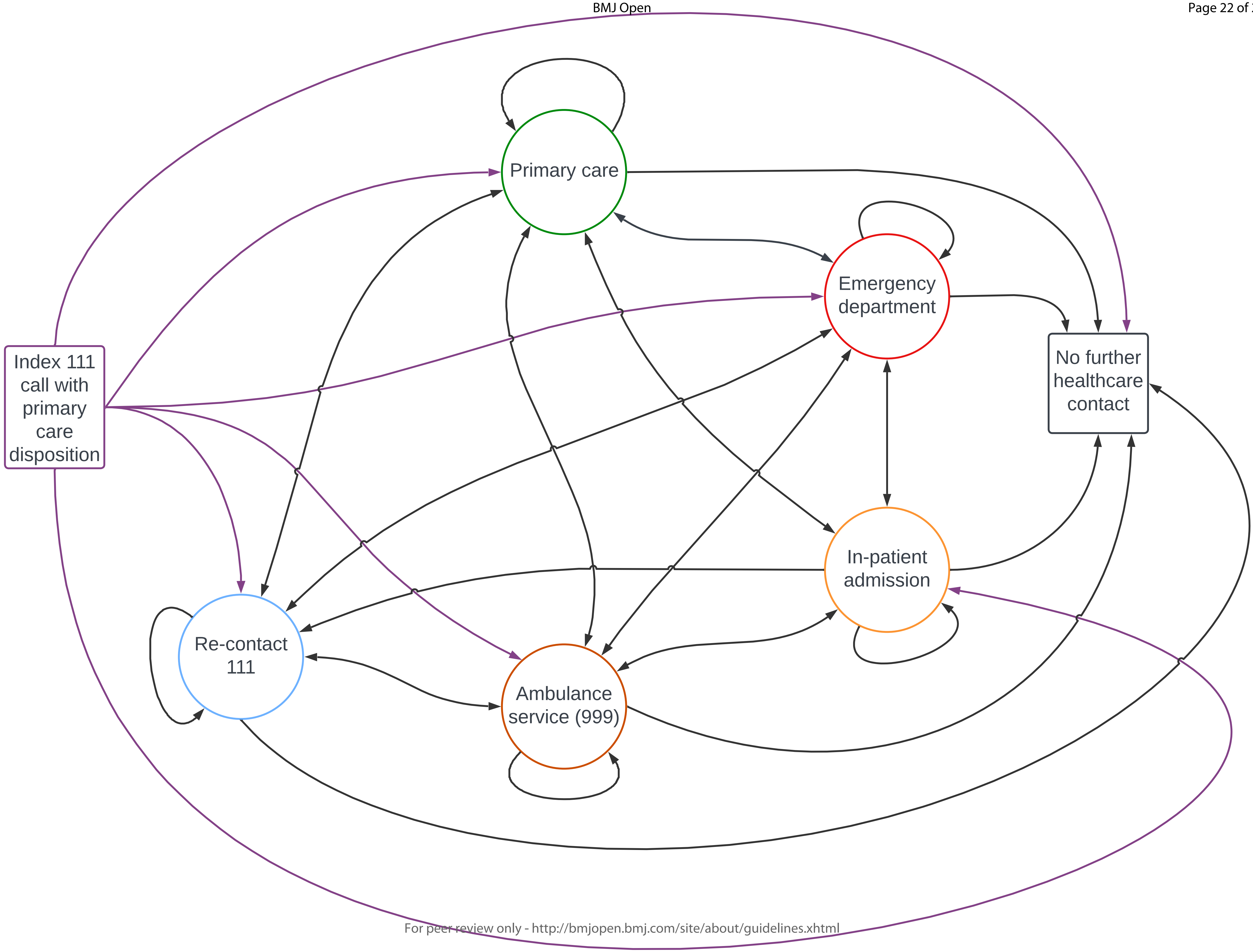
Primary care
Emergency department
No further healthcare contact
Index 111 call with primary care disposition
In-patient admission
Re-contact 111
Ambulance service (999)

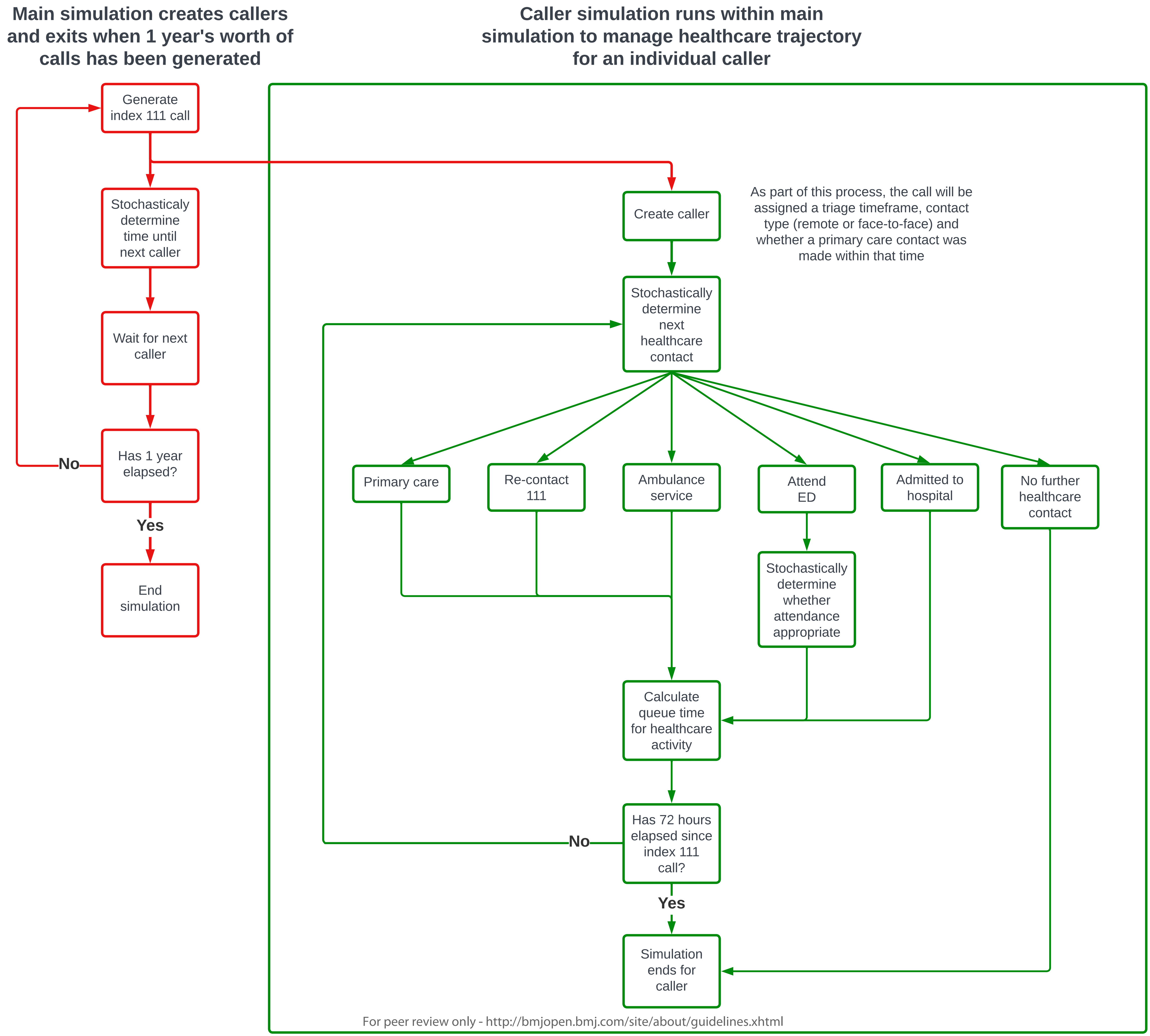
Main simulation creates callers and exits when 1 year's worth of calls has been generated
Caller simulation runs within main simulation to manage healthcare trajectory for an individual caller
Generate index 111 call
Stochasticaly determine time until next caller
Wait for next caller
Has 1 year elapsed?
No
Yes
End simulation
Create caller
As part of this process, the call will be assigned a triage timeframe, contact type (remote or face-to-face) and whether a primary care contact was made within that time
Stochastically determine next healthcare contact
Primary care
Re-contact 111
Ambulance service
Attend ED
Admitted to hospital
No further healthcare contact
Stochastically determine whether attendance appropriate
Calculate queue time for healthcare activity
Has 72 hours elapsed since index 111 call?
No
Yes
Simulation ends for caller

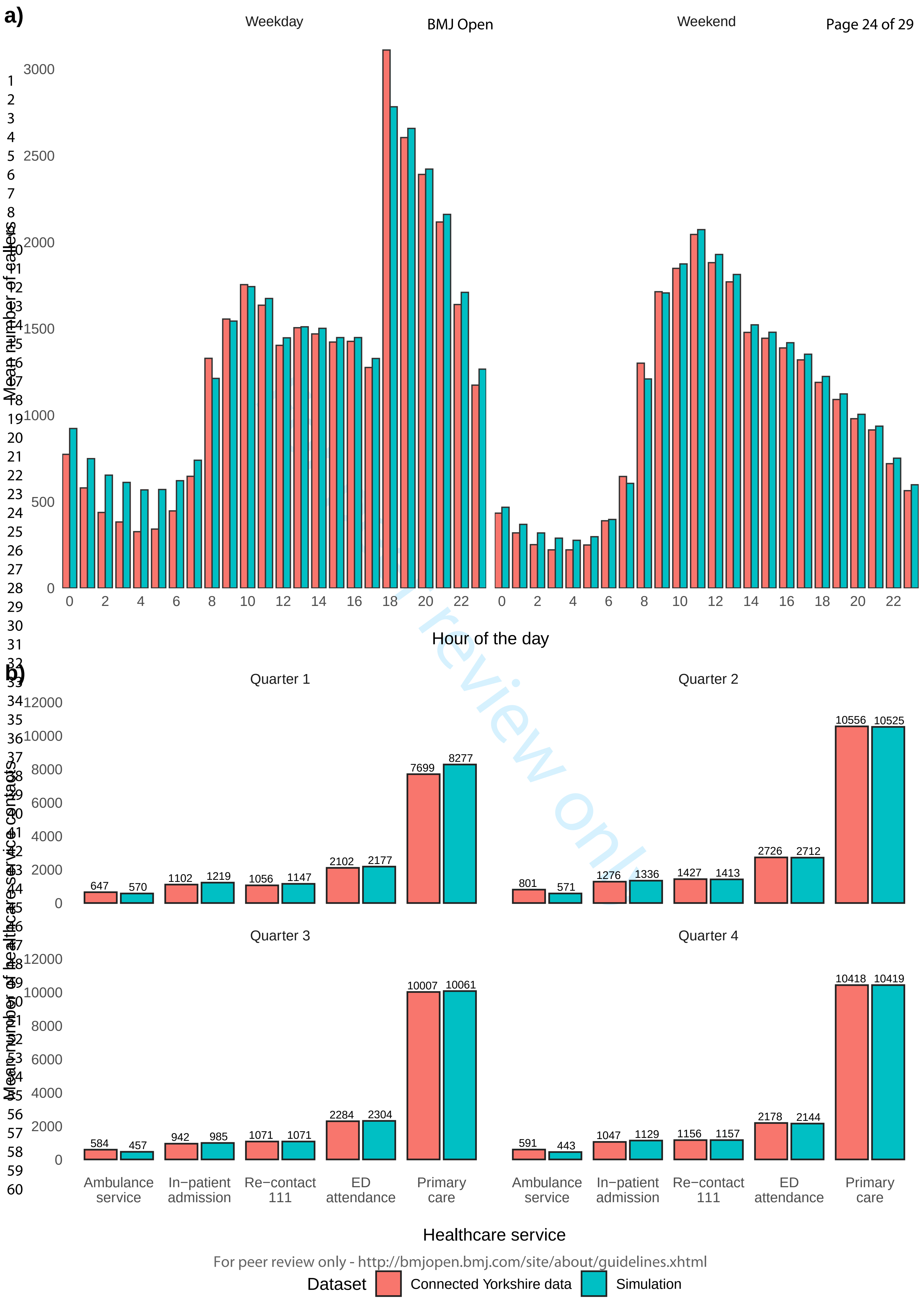
a)
Weekday
Weekend
Mean number of callers
Hour of the day
b)
Quarter 1
Quarter 2
Quarter 3
Quarter 4
Mean number of healthcare service contacts
647
570
1102
1219
1056
1147
2102
2177
7699
8277
801
571
1276
1336
1427
1413
2726
2712
10556
10525
584
457
942
985
1071
1071
2284
2304
10007
10061
591
443
1047
1129
1156
1157
2178
2144
10418
10419
Ambulance service
In−patient admission
Re−contact 111
ED attendance
Primary care
Healthcare service
Dataset
Connected Yorkshire data
Simulation

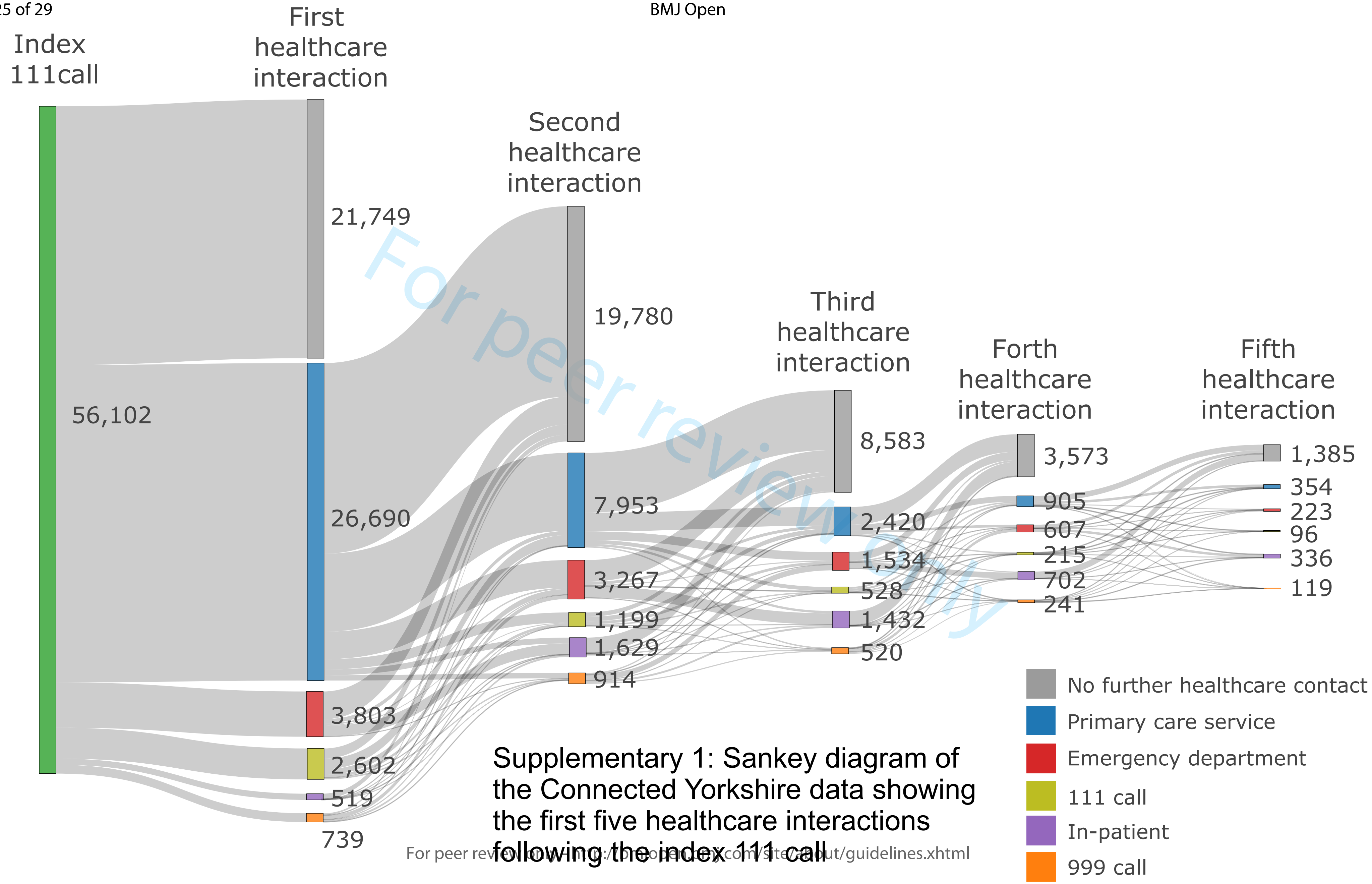

Supplementary 1: Sankey diagram of the Connected Yorkshire data showing the first five healthcare interactions following the index 111 call

For peer review only

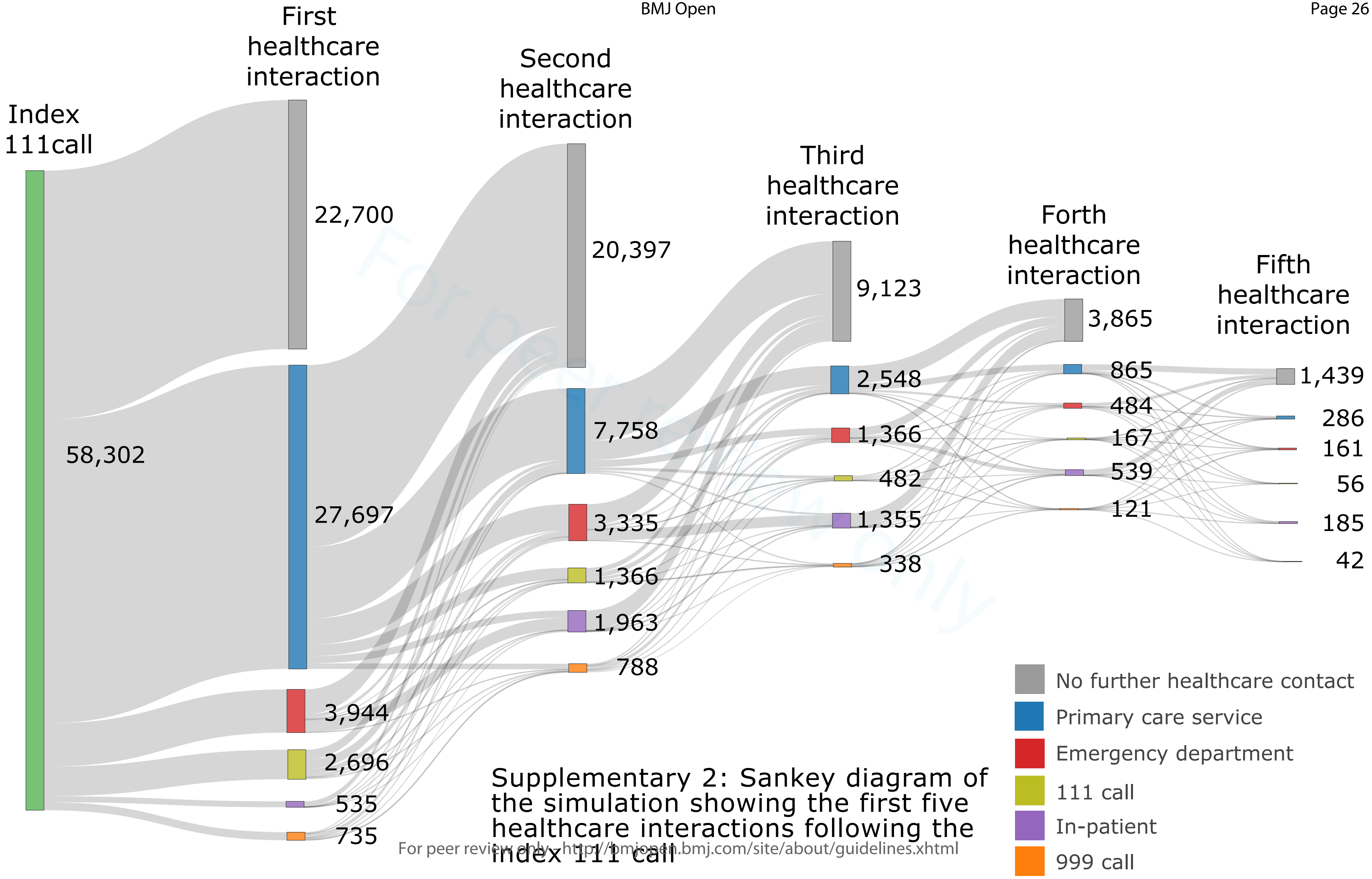

Supplementary 2: Sankey diagram of the simulation showing the first five healthcare interactions following the index 111 call

For peer review only - http://bmjopen.bmj.com/site/about/guidelines.xhtml

STROBE extension for simulation based research checklist

Item	Item no.	STROBE item	Location	Extension for SBR	Location
Title and abstract	1	a. Indicate the study's design with a commonly used term in the title or the abstract.<br>b. Provide in the abstract an informative and balanced summary of what was done and what was found.	a. Title<br><br>b. Abstract P2	In abstract or key terms, the MESH or searchable keyword term must have the word simulation or simulated.	Keywords: P1
Introduction					
Background/rationale	2	Explain the scientific background and rationale for the investigation being reported.	P2	Clarify whether simulation is subject of research or investigational method for research.	P2
Objectives	3	State specific objectives, including any prespecified hypotheses.	P2		
Methods					
Study design	4	Present key elements of study design early in the paper.	Methods		
Setting	5	Describe the setting, locations, and relevant dates, including periods of recruitment, exposure, follow-up, and data collection.	Data section		
Participants	6	a. Cohort study: give the eligibility criteria and the sources and methods of selection of participants. Describe methods of follow-up.<br>b. Cohort study: for matched studies, give matching criteria and number of exposed and unexposed.	a. Data section<br><br>b. N/A		
Variables	7	Clearly define all outcomes, exposures, predictors, potential confounders, and effect modifiers.<br>Give diagnostic criteria, if applicable.		Describe the theoretical and/or conceptual rationale for the design of the intervention/exposure.<br>Describe the intervention/exposure with sufficient detail to permit	Methods section: Conceptual modelling DES

STROBE extension for simulation based research checklist

				replication.<br>Clearly describe all simulation-specific exposures, potential confounders, and effect modifiers.	
Data sources/ measurement	8	For each variable of interest, give sources of data and details of methods of assessment (measurement).<br>Describe comparability of assessment methods if there is >1 group.	Data section	In describing the details of methods of assessment, include (when applicable) the setting, instrument, simulator type, timing in relation to the intervention, along with any methods used to enhance the quality of measurements.<br>Provide evidence to support the validity and reliability of assessment tools in this context (if available).	DES Analysis
Bias	9	Describe any efforts to address potential sources of bias.	Strengths and weaknesses		
Study size	10	Explain how the study size was arrived.			
Quantitative variables	11	Explain how quantitative variables were handled in the analyses. If applicable, describe which groupings were chosen and why.			
Statistical methods	12	a. Describe all statistical methods, including those used to control for confounding.<br>b. Describe any methods used to examine subgroups and interactions.<br>c. Explain how missing data were addressed.<br>d. Cohort study: if applicable, explain how loss to follow-up was addressed.<br>Case–control study: if applicable, explain how matching of cases and controls was addressed.<br>Cross-sectional study: if applicable, describe analytical methods taking	a. Methods<br>b. Methods<br>c. N/A<br>d. N/A	Clearly indicate the unit of analysis (e.g., individual, team, system), identify repeated measures on subjects, and describe how these issues were addressed.	

STROBE extension for simulation based research checklist

		account of sampling strategy.<br>e. Describe any sensitivity analyses.	e. N/A		
Results					
Participants	13	a. Report the numbers of individuals at each stage of the study (e.g., numbers potentially eligible, examined for eligibility, confirmed eligible, included in the study, completing follow-up, and analyzed).<br>b. Give reasons for nonparticipation at each stage.<br>c. Consider use of a flow diagram.	Sankey diagram (kind of).		
Descriptive data	14	a. Give characteristics of study participants (e.g., demographic, clinical, social) and information on exposures and potential confounders.<br>b. Indicate the number of participants with missing data for each variable of interest.<br>c. Cohort study: summarize follow-up time (e.g., average and total amount).	Results	In describing characteristics of study participants, include their previous experience with simulation and other relevant features as related to the intervention(s).	N/A
Outcome data	15	Cohort study: report numbers of outcome events or summary measures over time.<br>Case–control study: report numbers in each exposure category or summary measures of exposure.<br>Cross-sectional study: report numbers of outcome events or summary measures.	Results		
Main results	16	a. Give unadjusted estimates and, if applicable, confounder-adjusted estimates and their precision (e.g., 95 % confidence intervals).<br>Make clear which	A. Table 1, 2, 3	For assessments involving >1 rater, interrater reliability should be reported.	N/A

STROBE extension for simulation based research checklist

		confounders were adjusted for and why they were included.<br>b. Report category boundaries when continuous variables were categorized.<br>c. If relevant, consider translating estimates of relative risk into absolute risk for a meaningful time period.	b. N/A<br><br>c. N/A		
Other analyses	17	Report other analyses done (e.g., analyses of subgroups and interactions and sensitivity analyses).	Results		
Discussion					
Key results	18	Summarize key results with reference to study objectives.	First 2 paragraphs of Discussion		
Limitations	19	Discuss limitations of the study, taking into account sources of potential bias or imprecision. Discuss both direction and magnitude of any potential bias.	Strengths and weakness	Specifically discuss the limitations of SBR.	Strengths and weakness
Interpretation	20	Give a cautious overall interpretation of results considering objectives, limitations, multiplicity of analyses, results from similar studies, and other relevant evidence.	Discussion		
Generalisability	21	Discuss the generalisability (external validity) of the study results.	Strengths and weaknesses	Describe generalisability of simulation-based outcomes to patient-based outcomes (if applicable).	Strengths and weaknesses
Other information					
Funding	22	Give the source of funding and the role of the funders for the present study and, if applicable, for the original study on which the present article is based.	Funding	List simulator brand and if conflict of interest for intellectual property exists.	Methods – DES

BMJ Open

Modelling NHS England 111 demand for primary care services: a discrete event simulation

Journal:	*BMJ Open*
Manuscript ID	bmjopen-2023-076203.R2
Article Type:	Original research
Date Submitted by the Author:	21-Aug-2023
Complete List of Authors:	Pilbery, Richard; Yorkshire Ambulance Service NHS Trust, Research and Development<br>Smith, Madeleine; NHS Devon<br>Green, Jonathan; University of Plymouth Faculty of Health<br>Chalk, Daniel; University of Exeter Medical School,<br>O'Keeffe, Colin; The University of Sheffield, School of Health and Related Research
**Primary Subject Heading**:	Health informatics
Secondary Subject Heading:	Health services research
Keywords:	Primary Care < Primary Health Care, Telemedicine < BIOTECHNOLOGY & BIOINFORMATICS, Health informatics < BIOTECHNOLOGY & BIOINFORMATICS

SCHOLARONE™
Manuscripts

I, the Submitting Author has the right to grant and does grant on behalf of all authors of the Work (as defined in the below author licence), an exclusive licence and/or a non-exclusive licence for contributions from authors who are: i) UK Crown employees; ii) where BMJ has agreed a CC-BY licence shall apply, and/or iii) in accordance with the terms applicable for US Federal Government officers or employees acting as part of their official duties; on a worldwide, perpetual, irrevocable, royalty-free basis to BMJ Publishing Group Ltd ("BMJ") its licensees and where the relevant Journal is co-owned by BMJ to the co-owners of the Journal, to publish the Work in this journal and any other BMJ products and to exploit all rights, as set out in our licence.

The Submitting Author accepts and understands that any supply made under these terms is made by BMJ to the Submitting Author unless you are acting as an employee on behalf of your employer or a postgraduate student of an affiliated institution which is paying any applicable article publishing charge ("APC") for Open Access articles. Where the Submitting Author wishes to make the Work available on an Open Access basis (and intends to pay the relevant APC), the terms of reuse of such Open Access shall be governed by a Creative Commons licence – details of these licences and which Creative Commons licence will apply to this Work are set out in our licence referred to above.

Other than as permitted in any relevant BMJ Author's Self Archiving Policies, I confirm this Work has not been accepted for publication elsewhere, is not being considered for publication elsewhere and does not duplicate material already published. I confirm all authors consent to publication of this Work and authorise the granting of this licence.

For peer review only

Modelling NHS England 111 demand for primary care services: a discrete event simulation

Author information

Richard Pilbery (corresponding author)
Research paramedic
Yorkshire Ambulance Service NHS Trust
Springhill, Brindley Way
Wakefield 41 Business Park
Wakefield
WF2 0XQ
ORCID: https://orcid.org/0000-0002-5797-9788
email: r.pilbery@nhs.net

Madeleine Smith
NHS Devon

Jonathan Green
University of Plymouth
ORCID: https://orcid.org/0000-0001-5738-7515

Dr. Daniel Chalk
University of Exeter
ORCID: https://orcid.org/0000-0002-4165-4364

Colin O'Keeffe
University of Sheffield
ORCID: https://orcid.org/0000-0002-4484-7401

Keywords: 111, Telephone triage, primary care, emergency care, discrete event simulation

For peer review only

ABSTRACT

Objectives

This feasibility study aimed to model *in-silico* the current healthcare system for patients triaged to a primary care disposition following a call to NHS 111, and determine the effect of reconfiguring the healthcare system to ensure a timely primary care service contact.

Design

Discrete event simulation.

Setting

Single English NHS 111 call centre in Yorkshire.

Participants

Callers registered with a Bradford general practitioner (GP) who contacted the NHS 111 service in 2021 and were triaged to a primary care disposition.

Primary and secondary outcome measures

Face validity of conceptual model. Comparison between real and simulated data for quarterly counts (and 95% confidence intervals) for patient contact with emergency ambulance (999), 111, and primary and secondary care services. Mean difference and 95% confidence intervals in healthcare system usage between simulations and difference in mean proportion of avoidable admissions for callers who presented to an emergency department (ED).

Results

The simulation of the current system estimated that there would be 39,283 (95%CI 39,237–39,328) primary care contacts, 2,042 (95%CI 2,032–2,051) 999 calls and 1,120 (95%CI 1,114–1,127) avoidable ED attendances. Modifying the model to ensure a timely primary care response resulted in a mean percentage increase of 196.1% (95% CI 192.2–

For peer review only - http://bmjopen.bmj.com/site/about/guidelines.xhtml

199.9%) in primary care contacts, and a mean percentage decrease of 78.0% (95% CI 69.8–86.2%) in 999 calls and 88.1% (95% CI 81.7–94.5%) in ED attendances. Avoidable ED attendances reduced by a mean of -26 (95%CI -35– -17).

Conclusion

In this simulated study, ensuring timely contact with a primary care service would lead to a significant reduction in 999 and 111 calls, and ED attendances (although not avoidable ED attendance). However, this is likely to be impractical given the need to almost double current primary care service provision. Further economic and qualitative research is needed to determine whether this intervention would be cost effective and acceptable to both patients and primary care clinicians.

For peer review only

STRENGTHS AND LIMITATIONS OF THIS STUDY

- This study benefits from utilisation of a robust method (discrete event simulation) that has been widely used and accepted as a method to simulate healthcare system changes
- The model was informed by relevant stakeholders and a linked dataset.
- The model and its source code has been made available free and open source for others to interrogate and develop
- It is a simple working model, and does not take account of factors that have previously been associated with inappropriate ED attendance, such as age, sex, ethnicity and clinical input into the 111 call
- The data that informed the model only covers a discrete region in England, which may affect generalisability.

INTRODUCTION

The National Health Service (NHS) 111 service aims to assist members of the public with urgent medical care needs and is the successor to the NHS Direct service in England. Following pilots in four sites it was rolled out nationally, with the final site going live in

For peer review only - http://bmjopen.bmj.com/site/about/guidelines.xhtml

England in 2014, and in 2019/20 received over 19 million calls (1). Its key founding objective was to provide easy access to support for the public with urgent care needs, to ensure they received the "right care, from the right person, in the right place, at the right time" (2). It is also the key component of the 24/7 Integrated Urgent Care Service outlined in the NHS Long Term Plan (3).

The triage outcome of approximately 55% of calls made to the English National Health Service (NHS) 111 call service, is a referral to a primary care service. The triage process includes the method of contact (for example, callers may be asked to contact a primary care service themselves, or a referral made by 111 to a primary care service to contact the patient directly), and a timeframe within which this should occur. If a timely service (defined as a primary care contact within the time specified as part of the triage outcome) cannot be provided to patients, it is possible that this will result in patients calling 999 or attending emergency departments (ED) directly. We have previously reported that just under half (47.6%) of callers to 111 triaged to a primary care service disposition made contact with a primary care service as their first healthcare interaction (4). There was evidence that patients more frequently attended an ED when they had not had previous contact with a primary care service. However, rates of avoidable attendance were similar whether callers had contact with a primary care service prior to attendance at ED or not.

Other studies examining caller behaviour and the impacts on the wider healthcare system (5,6), have utilised datasets from 2017 and earlier, and patterns of activity identified then, may not be applicable now.

This study aimed to develop a Discrete Event Simulation to model the current healthcare system for callers triaged to a primary care disposition. Discrete Event Simulation models allow for the simulation of queuing problems, in which entities (such as patients) queue for processes that require resources (7). Our primary objective was to develop a model which represented the current healthcare system in relation to callers to 111 who received a primary care service disposition following triage. Our secondary objective was to use the model to estimate the predicted impact and associated resourcing required to ensure a timely primary care contact for all callers to the 111 service in a region of Yorkshire.

For peer review only

For peer review only - http://bmjopen.bmj.com/site/about/guidelines.xhtml

METHODS

Conceptual modelling

Prior to developing a simulation that represents the trajectories of patients within the model, it is necessary to develop a conceptual model: an appropriately simplified collection of assumptions about the components and structure of the 'real-world' (8) that can act as a blue print for the in-silico model development. This technique has its origins in soft systems methodology (9), and enabled us to work with stakeholders in the ambulance service, NHS 111, general practice and emergency departments to co-produce processes and capture their knowledge of the health and social care system. This co-production is not only crucial in terms of improving the accuracy of the model, but also an important component of stakeholder engagement and the formation of strategic partnerships (10).

Data

In tandem with the conceptual modelling phase, we obtained routine, retrospective data from the Connected Yorkshire research database, which provides linked patient-level data for approximately 1.2 million citizens across the Bradford and Airedale region of Yorkshire. Datasets include 111 and emergency ambulance (999) call data, as well as primary and secondary care (including emergency department and in-patient activity). Data submitted to Connected Yorkshire contain a unique identifier in the form of a pseudonymised NHS number (used throughout the NHS as a unique identifier for patient care). In this study, we used it, along with the date and time of access to services, to track a caller's healthcare journey.

We identified all 111 calls between the 1st January 2021 and 31st December 2021 for patients who were triaged to a primary care disposition and registered with a General Practitioner (family physician, GP) in the Bradford area at the time of the call. Subsequent healthcare system access in the 72 hours following the first (index) call, was identified by programmatically searching for the patient's unique identifier in the 111, 999 calls, primary care, hospital emergency department and in-patient admission datasets (4).

For peer review only - http://bmjopen.bmj.com/site/about/guidelines.xhtml

Triage to a primary care service in this study refers to a range of services, including referral to the patient's own GP, or an out-of-hours GP service, pharmacy, community, optician, mental health or maternity service. Depending on the triage disposition and service availability, referrals are either made directly by 111 with a request for a service to contact the caller, or the caller is advised to contact/attend the service directly. In the source data, over 96% of primary care disposition referrals, were to a GP (or out-of-hours service) and of the other services, only pharmacies, mental health and optician service interactions would not appear in the data. However, these services only comprised 2% of triage outcomes in the source data (4).

Discrete event simulation

Using (11) taxonomy, we determined that Discrete Event Simulation (DES) was a good choice of operational research methodology to address the research question. DES is a flexible modelling technique that can represent complex behaviour of callers to 111, and their interactions with the healthcare system simultaneously. DES can also incorporate capacity and resource constraints and account for chance, for example the stochastic behaviour of callers (7,7,12). DES is also useful, because it makes it possible to ask 'what if' questions such as the research question. It has been used widely in healthcare, examining health care systems operations, and disease progression, screening and health behaviour modelling (7,13).

We created an *in-silico* version of the conceptual model using the programming language Python (v3.9.6) and the SymPy library (v4.0.1, 14). In addition, we created a web-based dashboard in order to present the results of the simulation and provide an opportunity for users/stakeholders to run their own simulations. To achieve this, we used Plotly Dash (v5.11.0, 15) and created a fully contained environment in docker (v4.2.0.7078) which is available from the study GitHub repository (https://github.com/RichardPilbery/MOOOD-study).

Patients enter the model at the point they are triaged to a primary care disposition by 111 and have exited the service. The 'arrival' rate of callers into the model is determined by sampling from an exponential probability distribution with lambda set to the 1/mean

For peer review only - http://bmjopen.bmj.com/site/about/guidelines.xhtml

arrival rate for 111 calls, stratified by hour, yearly quarter and whether the call occurred at a weekend. When a patient is created in the simulation, they are assigned a primary care disposition with an associated triage acuity (Figure 1). This consists of a time frame within which a contact with a primary care service should be made, and specifies whether the contact should be face-to-face or remotely, for example by telephone. The allocation is random, but weighted according to the proportions seen in the Connected Yorkshire data. Once the patient has been allocated a primary care disposition, the uniform distribution is sampled to determine whether the primary care contact will be achieved in the specified triage time frame. This is assumed to be the case when the sampled value is less than proportion calculated from the Connected Yorkshire dataset.

Patient trajectory through the healthcare system is determined using transition probabilities calculated from the Connected Yorkshire dataset using the statistics software, R (16). The next health care service (including no further contact) is determined by random sampling, weighted by whether a primary care service contact was made immediately after the index 111 call, and the yearly quarter. If the next healthcare service access is determined to be the emergency department (ED), then the uniform distribution is sampled to determine whether the admission will be avoidable. As before, this is assumed to be the case when the sampled value is less than proportion calculated from the Connected Yorkshire dataset. The proportions are stratified by primary care disposition and whether initial contact was made with a primary care service.

For this study, an ED attendance is classed as being 'avoidable' when it meets the O'Keeffe et al (17) criteria. They defined an avoidable attendance as a patient presenting to a consultant-led ED which provides a 24 hour service with full resuscitation facilities and designated accommodation for the reception of emergency care patients (referred to as a type 1 ED (18)), but who do not receive investigations, treatments or referral that required the facilities of a type 1 ED.

Unless the patient is allocated to 'no further healthcare contact', in which case the patient exits the simulation, the 'queue' time i.e. the time the patient waits until the service is accessed and the time that the healthcare activity takes, is calculated. The 'queue' time is

For peer review only - http://bmjopen.bmj.com/site/about/guidelines.xhtml

calculated by sampling from a distribution specific to the current healthcare service being access and the next, for example index 111 call to ED attendance. Activity times are calculated by sampling from distributions specific to the service being accessed. In both cases, the optimal distribution was chosen by the Python library fitter (v1.5.2, 19) based on the distribution with the lowest sum of square errors.

Patients remain in the simulation for a maximum of 72 hours unless they are allocated to 'no further healthcare contact' prior to this. The model generates 111 calls for a period of 1 year of simulated time.

Analysis

Once the model was developed, we performed 100 runs of 1 year's worth of simulated 111 calls and monitored the patient's trajectory over the subsequent 72 hours. As the model is stochastic, multiple runs of the model were necessary to ensure that results are representative and not due to stochastic noise. A summary of all simulations was reported as the mean and 95% confidence intervals for counts and proportions.

Since all models are approximations of the 'real world', we used the historic data provided by the Connected Yorkshire dataset to see how closely our model matches with real data (the primary objective), visually and descriptively analysing the difference in temporal distribution of calls between the actual and simulated data, as well as comparing quarterly aggregated healthcare service access by patients, following the index 111 call. Finally, we performed a visual assessment of patient trajectory, to determine whether it approximated actual patient behaviour.

For the secondary objective, we undertook the 'what if' analysis, examining the hypothetical situation whereby all index 111 calls with a primary care disposition received a timely (i.e. within the specified call triage time) response from a primary care service. No capacity constraints were placed on primary care services in order to estimate the true resourcing that would be required to meet the demand appropriately. We calculated the mean difference and 95% confidence intervals in healthcare system usage between

For peer review only - http://bmjopen.bmj.com/site/about/guidelines.xhtml

simulations and determined the difference in mean proportion of avoidable admissions for callers who presented to an ED.

Patient and Public Involvement

The application and protocol for this study was review by the Yorkshire Ambulance Service NHS Trust patient research ambassador. In addition, Connected Bradford have an active patient and public involvement group and a representative attended the approvals board for this study.

RESULTS

We visually compared outputs from the model with historic patient data and observed sufficient similarity in the distribution pattern of calls across the day, which gave us confidence that the model was sufficiently capturing the variability within the real-world data (Figure 2, Figure 3). 111 call activity in both simulations was similar to the actual call activity seen in the real data, although overall, the simulation did slightly overestimate the number of calls, particularly between 00:00 and 06:00 (Figure 2a). The simulation tended to underestimate 999 call activity and overestimate all other healthcare service activity. Primary care, ED admission and subsequent 111 call activity was most closely simulated (Table 1). Primary care, ED attendance and subsequent 111 call activity was most closely simulated (Figure 2b, Figure 3a).

Table 1: Comparison of total healthcare service contacts between the origin simulation and 'what if?' scenario

Service	Connected Yorkshire data (N)	Simulation (mean, 95%CI)	What if? (mean, 95%CI)	Mean difference (mean 95%CI)	Mean percentage change (mean %, 95%CI)
Ambulance service	2,623	2,042 (2,032–	1,593 (1,584–	-449 (-461–-436)	78 (69.8–86.2)

For peer review only - http://bmjopen.bmj.com/site/about/guidelines.xhtml

Service	Connected Yorkshire data (N)	Simulation (mean, 95%CI)	What if? (mean, 95%CI)	Mean difference (mean 95%CI)	Mean percentage change (mean %, 95%CI)
		2,051)	1,602)		
Emergency department	9,290	9,337 (9,319–9,355)	8,228 (8,213–8,244)	-1,109 (-1,133– -1,085)	88.1 (81.7–94.5)
Primary care	38,680	39,283 (39,237–39,328)	77,030 (76,964–77,097)	37,748 (37,667–37,829)	196.1 (192.2–199.9)
In-patient	4,367	4,668 (4,654–4,681)	4,620 (4,607–4,632)	-48 (-66– -30)	99 (97-- 101)
111	4,710	4,789 (4,771–4,806)	3,074 (3,061–3,087)	-1,715 (-1,737– -1,693)	64.2 (54.7–ß 73.7)

The first healthcare contact following the index 111 call in the Connected Yorkshire data was most commonly a primary care service (26,690/56102; 47.6%). However, a large proportion (21,749/56,102; 38.8%) had no healthcare contact in the 72 hours following the index 111 call. This was mirrored by the simulation data, where the first contact following the index 111 call was a primary care service in a mean of 27,697/58,302 (47.5%) of cases, with no further healthcare contact the second most common finding (22,700/58,302; 38.9%) (Figure 3b).

"What if?" scenario

We simulated a hypothetical scenario whereby all 111 calls with a primary care disposition received a timely primary care contact (the 'what if?' scenario). This assumed that there

For peer review only - http://bmjopen.bmj.com/site/about/guidelines.xhtml

were no capacity constraints on the service and that all callers accessed a primary care service following the index 111 call.

This resulted in an increase in primary care contacts from a mean of 39,283 (95%CI 39,237–39,328) in the base simulation to 77,030 (95%CI 76,964–77,097); a mean percentage increase of 196.1% (95%CI, 192.2– 199.9%) (Table 1). All other healthcare services saw a reduction in healthcare interactions, with the most notable reductions occurring in 999 and 111 service access (mean percentage change 78.0% and 64.2%, respectively).

ED attendance

In the original Connected Yorkshire dataset, there were 9,290 emergency department attendances and 1,029 (11.1%) met the O'Keefe et al (17) definition of an avoidable attendance. The simulated dataset estimated a mean ED attendance of 9,337 cases (95%CI 9,319–9,356), of which, 1,120 (95%CI 1,114–1,127; mean proportion, 12%, 95%CI 5.6–18.4%) were classed as avoidable. In the 'what if' scenario, ED attendances reduced to a mean of 8,228 (95%CI 8,213–8,244) attendances but there was little change in the proportion of avoidable attendance (mean proportion 13.3%, 95%CI 6.6–20%).

Discussion

Using a simple simulation model, we have been able to successfully capture the healthcare system as it relates to callers to 111 who are triaged to a primary care disposition. We used the model to estimate the impact of a hypothetical scenario in which primary care services had sufficient capacity to provide a service to all 111 callers triaged to their service and that all callers elected to contact the service. We determined that the 'base case' model sufficiently closely replicated the real-world data to be confident that its estimates of the impact of the scenario tested would be useful. While we did not monitor or simulate ambulance dispatch, we can still see the outcome of any physical attendance by an ambulance crew by monitoring ED attendances or GP contacts following a 999 call, for example.

For peer review only

In the simulated 'what if?' scenario, we found that ensuring a timely primary care contact for all 111 callers triaged to a primary care disposition, would lead to a significant reduction in ambulance service and 111 contacts and emergency department admissions. However, to achieve this it would be necessary to almost double the number of primary care contacts provided by the current healthcare system which, in the wider context of clinical staff shortages in general practice (20,21) and the NHS (22), is unlikely to be feasible. While we did not conduct an economic analysis, the data suggests that the savings made by reducing certain healthcare service contacts are unlikely to offset the additional cost in providing additional primary care services.

Even if the capacity issues could be overcome, adjusting caller behaviour could be even more challenging. Almost 39% of callers in the base case model did not seek any further healthcare contact in the 72 hours after the index 111 call. Previous qualitative work on understanding why patients call an ambulance for 'primary care problems' highlighted the perceived limitations of out-of-hours primary care services by patients to address their needs and, in the case of patients with chronic conditions, previous negative experiences of community-based healthcare services being unable to 'help' would need to be addressed (23). In addition, there are reasons why patients may not follow advice to contact primary care which are beyond the control of the health system, such as work or family/childcare commitments, transport issues (including weather-related) and demographic factors such as younger age, female sex and low socio-economic background (24); factors that would be unaffected by changes in service provision. That said, there is limited evidence that increasing availability of primary care services (such as offering extended hours) can lead to a reduction in access other services. This is particularly pertinent, since most 111 calls with a primary care disposition occur out-of-hours (25).

This work has generated stakeholder discussion about whether the apparent supply/demand problem identified by the simulation could be addressed by utilising the NHS Additional Roles Reimbursement Scheme which provides funding for healthcare professionals (HCP) to work as a multi-disciplinary team within primary care to expand capacity and support delivery of new services tailored to the local population. Potential

For peer review only

HCP roles include pharmacists, paramedics, occupational therapists, and dieticians in addition to others.

In addition, they have also suggested that further qualitative work might help to understand which primary care roles best suit particular caller presentations (for example a consultation with physiotherapist rather than a GP for a musculoskeletal presentation), which may reduce the proportion of callers who do not access healthcare services following the 111 call and are potentially left with an unmet healthcare need.

Strengths and weaknesses

This study benefits from utilisation of a robust method (discrete event simulation) that has been widely used and accepted as a method to simulate healthcare system changes (26). In addition, the model was informed by relevant stakeholders and a linked dataset. However, it is nevertheless a simple working model, and does not take account of factors that have previously been associated with inappropriate ED attendance, such as age, sex, ethnicity and clinical input into the 111 call (6).

The model may benefit from more extensive validation and verification than the simple 'black box' validation described here, in which outputs from the model were compared with the equivalent real world metrics. Additional validation measures, such as performing sensitivity analyses to determine the impact of inaccuracies in modelling assumptions could be useful in increasing confidence in the model's predictions (27). In addition, verification measures such as extensive review of code by others can help to identify issues in the software engineering side of the development (28). In addition, only callers who are registered with a Bradford GP were included. Given that GP registrations are consistently higher than Office for National Statistics (ONS) population estimates, it is difficult to determine what proportion of the population are not registered with a GP. Patients also have a right to opt-out of their clinical data being used for research purposes, and this comprises around 4% of patients registered with a GP in England (29).

The data that informed the model only covers a discrete region in West Yorkshire, which may affect generalisability. Bradford is mainly an urban area and the 13th most deprived

For peer review only - http://bmjopen.bmj.com/site/about/guidelines.xhtml

For peer review only

local authority in England (out of 333) based on the Index of Multiple Deprivation (30). In addition, it is based on data collected in 2021, during the pandemic, which may not be representative of the current healthcare trajectory for patients who call 111. However, the open source nature of the model means that others can use the model to parameterise according to the data from their own regions.

Further economic and qualitative analysis would be beneficial to determine whether an intervention such as this would be cost-effective, feasible and acceptable to patients. This could potentially include ambulance attendance following 999 calls, which may have implications for ambulance service operational performance and cost effectiveness.

CONCLUSION

In this simulated study, ensuring timely contact with a primary care service is estimated to lead to a significant reduction in 999 and 111 calls, and ED attendances (although not avoidable ED attendance). However, this is likely to be impractical given the estimated need to almost double current primary care service provision. Further economic and qualitative research is needed to determine whether this intervention would be cost effective and acceptable to both patients and primary care providers.

Acknowledgements

This work uses data provided by patients and collected by the NHS as part of their care and support. The authors would also like to thank Dr. Eithne Cummins and Dr. Jon Dickson for providing a stakeholder perspective to the study, and Dr. Hazel Squires for early support in operational research methodology. In addition, we are grateful for the support provided by the team at Connected Yorkshire, especially Kuldeep Sohal and John Birkinshaw.

The work was supported as a project of the NIHR Applied Research Collaboration for the South West Peninsula Health Service Modelling Associates (HSMA) Programme (https://sites.google.com/nihr.ac.uk/hsma) which also provided the training required in Python and Discrete Event Simulation.

For peer review only - http://bmjopen.bmj.com/site/about/guidelines.xhtml

Contributors

RP and DC conceived and designed the study. JG conducted a literature review. RP obtained the research approvals to access the datasets and acts as guarantor for the paper. RP and MS developed the code necessary to extract the data that informed the model. RP coded the model. DC provided mentoring support and guidance for the project, and delivered the training in Python and SimPy. CO assisted with the design of the study. All authors drafted the manuscript and contributed substantially to its revision.

Funding

This paper presents independent research by the NIHR Applied Research Collaboration Yorkshire and Humber (ARC YH). This work was supported by the National Institute for Health Research Applied Research Collaboration South West Peninsula and Yorkshire and Humber.

Disclaimer

The views expressed in this publication are those of the author(s) and not necessarily those of the National Institute for Health Research or the Department of Health and Social Care.

Data sharing

The complete model, including transition probabilities and inter-arrival, activity and queue distributions are available from the study GitHub repository (https://github.com/RichardPilbery/MOOOD-study). The software is supplied under a GNU General Public License.

Competing interests

The authors declare not completing interests with respect to this work.

For peer review only - http://bmjopen.bmj.com/site/about/guidelines.xhtml

Ethical approval

This study was approved by the Bradford Learning Health System Board in accordance with the Connected Yorkshire NHS Research Ethics Committee (REC) and Confidentiality Advisory Group (CAG) approvals relating to the Connected Yorkshire research database (17/EM/0254). No separate Health Research Authority (HRA) approval was required for this study.

REFERENCES

1. NHS England. NHS 111 Minimum Data Set 2019-20 [Internet]. 2020 [cited 2020 Dec 29]. Available from: https://www.england.nhs.uk/statistics/statistical-work-areas/nhs-111-minimum-data-set/nhs-111-minimum-data-set-2019-20/

2. UK Government. NHS 111 [Internet]. 2011 [cited 2020 Dec 10]. Available from: https://web.archive.org/web/20130415084921/http://www.connectingforhealth.nhs.uk/systemsandservices/pathways/news/nhs111intro.pdf

3. England N. The NHS Long Term Plan [Internet]. NHS Long Term Plan. 2019 [cited 2020 Dec 10]. Available from: https://www.longtermplan.nhs.uk/publication/nhs-long-term-plan/

4. Pilbery R, Smith M, Green J, Chalk D. An analysis of NHS 111 demand for primary care services [Internet]. medRxiv; 2023 [cited 2023 Apr 5]. Available from: https://www.medrxiv.org/content/10.1101/2023.03.20.23287449v1

5. Lewis J, Stone T, Simpson R, Jacques R, O'Keeffe C, Croft S, et al. Patient compliance with NHS 111 advice: Analysis of adult call and ED attendance data 2013–2017. PLOS ONE [Internet]. 2021 May [cited 2021 May 28];16(5):e0251362. Available from: https://journals.plos.org/plosone/article?id=10.1371/journal.pone.0251362

6. Nakubulwa MA, Greenfield G, Pizzo E, Magusin A, Maconochie I, Blair M, et al. To what extent do callers follow the advice given by a non-emergency medical helpline (NHS

For peer review only

For peer review only - http://bmjopen.bmj.com/site/about/guidelines.xhtml

111): A retrospective cohort study. Montefiori M, editor. PLOS ONE [Internet]. 2022 Apr [cited 2023 Mar 2];17(4):e0267052. Available from: https://dx.plos.org/10.1371/journal.pone.0267052

7. Vázquez-Serrano JI, Peimbert-García RE, Cárdenas-Barrón LE. Discrete-Event Simulation Modeling in Healthcare: A Comprehensive Review. International Journal of Environmental Research and Public Health [Internet]. 2021 Nov [cited 2023 Mar 8];18(22):12262. Available from: https://www.ncbi.nlm.nih.gov/pmc/articles/PMC8625660/

8. Kotiadis K, Tako AA, Vasilakis C. A participative and facilitative conceptual modelling framework for discrete event simulation studies in healthcare. Journal of the Operational Research Society [Internet]. 2014 Feb [cited 2023 Jul 27];65(2):197–213. Available from: https://doi.org/10.1057/jors.2012.176

9. Kotiadis K. Using soft systems methodology to determine the simulation study objectives. Journal of Simulation [Internet]. 2007 Aug [cited 2023 Jul 27];1(3):215–22. Available from: https://doi.org/10.1057/palgrave.jos.4250025

10. Franco LA, Montibeller G. Facilitated modelling in operational research. European Journal of Operational Research [Internet]. 2010 Sep [cited 2023 Jul 27];205(3):489–500. Available from: https://www.sciencedirect.com/science/article/pii/S0377221709006699

11. Brennan A, Chick SE, Davies R. A taxonomy of model structures for economic evaluation of health technologies. Health Economics [Internet]. 2006 Dec [cited 2019 Mar 7];15(12):1295–310. Available from: http://doi.wiley.com/10.1002/hec.1148

12. Karnon J, Stahl J, Brennan A, Caro JJ, Mar J, Möller J. Modeling Using Discrete Event Simulation: A Report of the ISPOR-SMDM Modeling Good Research Practices Task Force–4. Medical Decision Making [Internet]. 2012 Sep [cited 2018 Nov 18];32(5):701–11. Available from: https://doi.org/10.1177/0272989X12455462

For peer review only - http://bmjopen.bmj.com/site/about/guidelines.xhtml

13. Forbus JJ, Berleant D. Discrete-Event Simulation in Healthcare Settings: A Review. Modelling [Internet]. 2022 Dec [cited 2023 Mar 8];3(4):417–33. Available from: https://www.mdpi.com/2673-3951/3/4/27

14. Meurer A, Smith CP, Paprocki M, Čertík O, Kirpichev SB, Rocklin M, et al. SymPy: Symbolic computing in Python. PeerJ Computer Science [Internet]. 2017 Jan;3:e103. Available from: https://doi.org/10.7717/peerj-cs.103

15. Plotly Technologies Inc. Collaborative data science [Internet]. 2015 [cited 2023 Mar 17]. Available from: https://plot.ly

16. R Core Team. R: A language and environment for statistical computing [Internet]. Vienna, Austria; 2022. Available from: https://www.R-project.org/

17. O'Keeffe C, Mason S, Jacques R, Nicholl J. Characterising non-urgent users of the emergency department (ED): A retrospective analysis of routine ED data. PLOS ONE [Internet]. 2018 Feb [cited 2022 Jun 24];13(2):e0192855. Available from: https://journals.plos.org/plosone/article?id=10.1371/journal.pone.0192855

18. NHS Digital. Emergency care department type [Internet]. 2023 [cited 2023 Mar 14]. Available from: https://www.datadictionary.nhs.uk/attributes/emergency_care_department_type.html

19. Cokelaer T, Kravchenko A, Lahdjirayhan, Msat59, Varma A, L B, et al. Cokelaer/fitter: v1.5.2 [Internet]. Zenodo; 2023 [cited 2023 Mar 3]. Available from: https://zenodo.org/record/7497983

20. The Health Foundation. A quarter of GP and general practice nursing posts could be vacant in 10 years [Internet]. 2022 [cited 2023 Mar 13]. Available from: https://www.health.org.uk/news-and-comment/news/a-quarter-of-gp-and-general-practice-nursing-posts-could-be-vacant-in-10-years

21. British Medical Association. Pressures in general practice data analysis [Internet]. The British Medical Association is the trade union and professional body for doctors in the UK. 2023 [cited 2023 Mar 13]. Available from: https://www.bma.org.uk/advice-and-

For peer review only

support/nhs-delivery-and-workforce/pressures/pressures-in-general-practice-data-analysis

22. Waitzman E. Staff shortages in the NHS and social care sectors [Internet]. 2022 [cited 2023 Mar 13]. Available from: https://lordslibrary.parliament.uk/staff-shortages-in-the-nhs-and-social-care-sectors/

23. Booker MJ, Simmonds RL, Purdy S. Patients who call emergency ambulances for primary care problems: A qualitative study of the decision-making process. Emergency Medicine Journal [Internet]. 2014 Jun [cited 2023 Mar 18];31(6):448–52. Available from: https://emj.bmj.com/content/31/6/448

24. Parsons J, Bryce C, Atherton H. Which patients miss appointments with general practice and the reasons why: A systematic review. British Journal of General Practice [Internet]. 2021 Jun [cited 2023 Jan 13];71(707):e406–12. Available from: https://bjgp.org/content/71/707/e406

25. Whittaker W, Anselmi L, Kristensen SR, Lau YS, Bailey S, Bower P, et al. Associations between Extending Access to Primary Care and Emergency Department Visits: A Difference-In-Differences Analysis. PLOS Medicine [Internet]. 2016 Sep [cited 2023 Mar 10];13(9):e1002113. Available from: https://journals.plos.org/plosmedicine/article?id=10.1371/journal.pmed.1002113

26. Caro JJ, Möller J, Karnon J, Stahl J, Ishak J. Discrete event simulation for health technology assessment. Boca Raton ; London: CRC Press, Taylor & Francis Group; 2016.

27. Macdonald I, Strachan P. Practical application of uncertainty analysis. Energy and Buildings [Internet]. 2001 Feb [cited 2023 Apr 12];33(3):219–27. Available from: https://www.sciencedirect.com/science/article/pii/S0378778800000852

28. Raunak M, Olsen M. Quantifying validation of discrete event simulation models. In: Proceedings of the Winter Simulation Conference 2014. 2014. p. 628–39.

For peer review only - http://bmjopen.bmj.com/site/about/guidelines.xhtml

29. NHS Digital. [MI] National Data Opt-out, May 2023 [Internet]. NDRS. 2023 [cited 2023 Jul 19]. Available from: https://digital.nhs.uk/data-and-information/publications/statistical/national-data-opt-out/may-23

30. City of Bradford Metropolitan District Council. Indices of Deprivation 2019 - Intelligence bulletin [Internet]. 2019 [cited 2023 Mar 15]. Available from: https://ubd.bradford.gov.uk/media/1534/indices-of-deprivation-2019-intelligence-bulletin.pdf

For peer review only

FIGURE LEGENDS

- Figure 1: Programmatic simulation flow chart
- Figure 2: a) Comparison between Connected Yorkshire and simulation data of hourly caller numbers stratified by weekday/weekend. b) Comparison between Connected Yorkshire and simulation data of healthcare service contacts, stratified by service and yearly quarter.
- Figure 3: a) Sankey diagram of the Connected Yorkshire data showing the first five healthcare interactions following the index 111 call. b) Sankey diagram of the simulation showing the first five healthcare interactions following the index 111 call

For peer review only - http://bmjopen.bmj.com/site/about/guidelines.xhtml

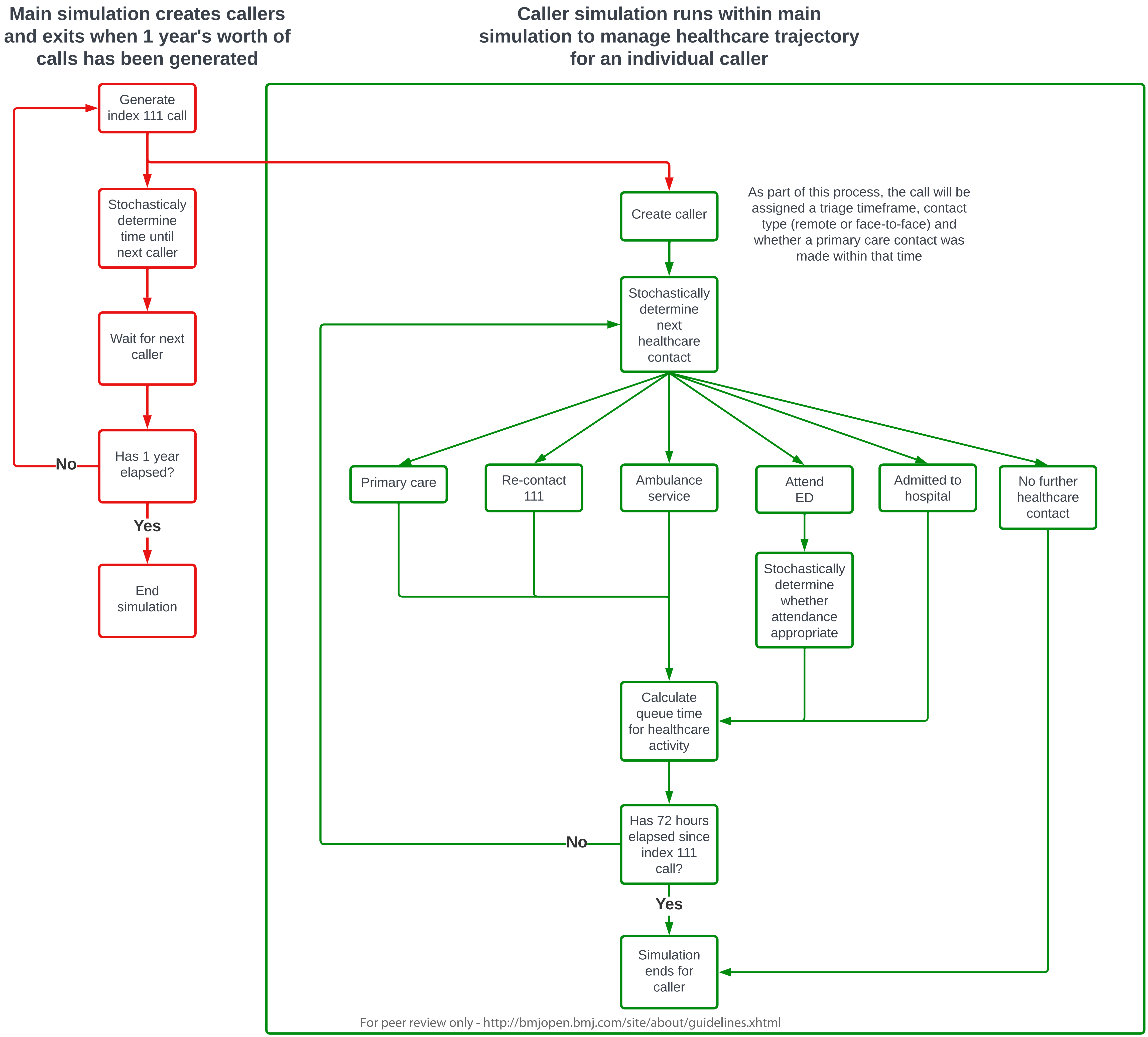

For peer review only - http://bmjopen.bmj.com/site/about/guidelines.xhtml

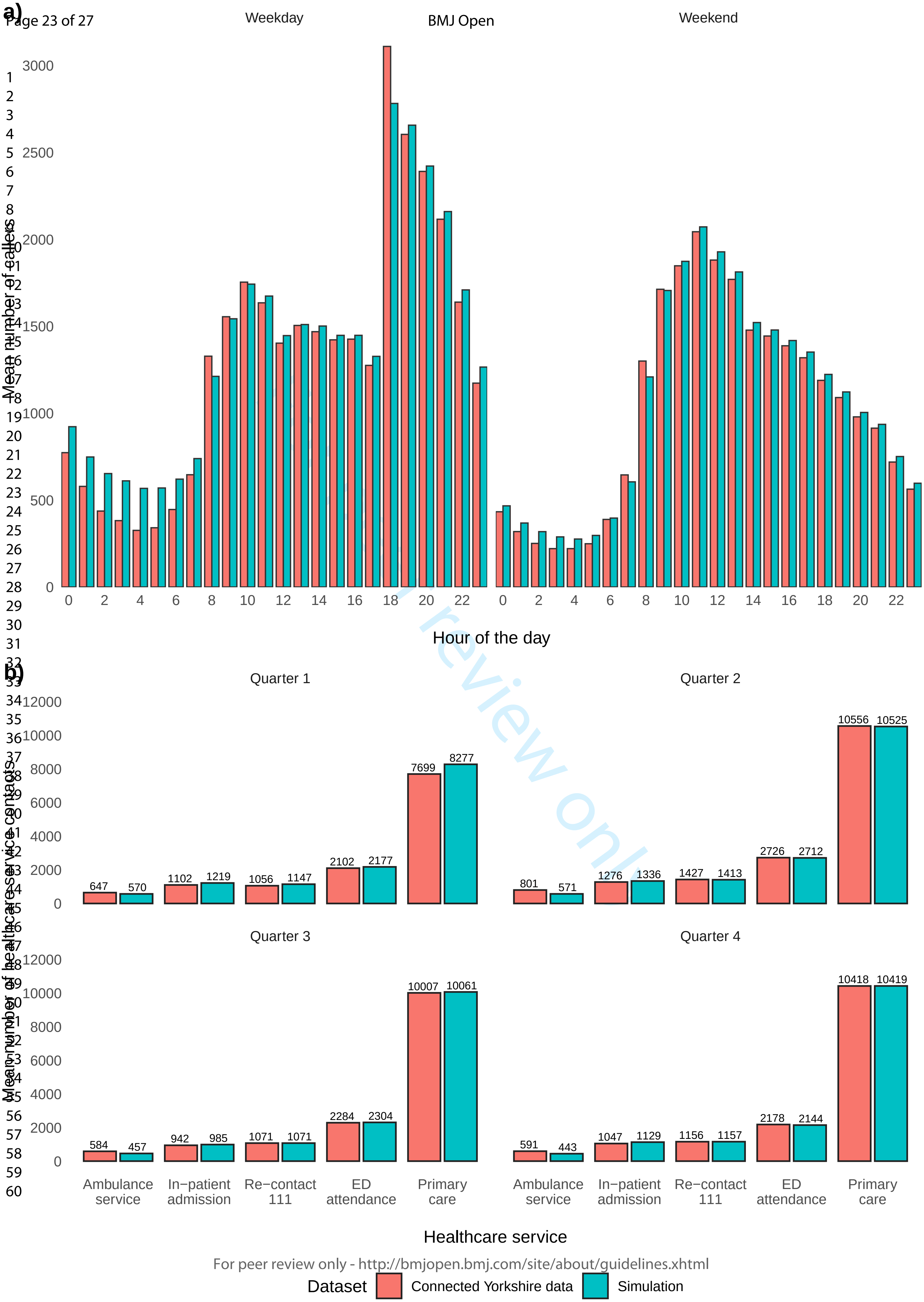
a)
Weekday
Weekend
Mean number of callers
3000
2500
2000
1500
1000
500
0
0 2 4 6 8 10 12 14 16 18 20 22
Hour of the day
b)
Quarter 1
Quarter 2
Quarter 3
Quarter 4
Mean number of healthcare service contacts
12000
10000
8000
6000
4000
2000
0
647 570 1102 1219 1056 1147 2102 2177 7699 8277
801 571 1276 1336 1427 1413 2726 2712 10556 10525
584 457 942 985 1071 1071 2284 2304 10007 10061
591 443 1047 1129 1156 1157 2178 2144 10418 10419
Ambulance service
In−patient admission
Re−contact 111
ED attendance
Primary care
Healthcare service
Dataset
Connected Yorkshire data
Simulation

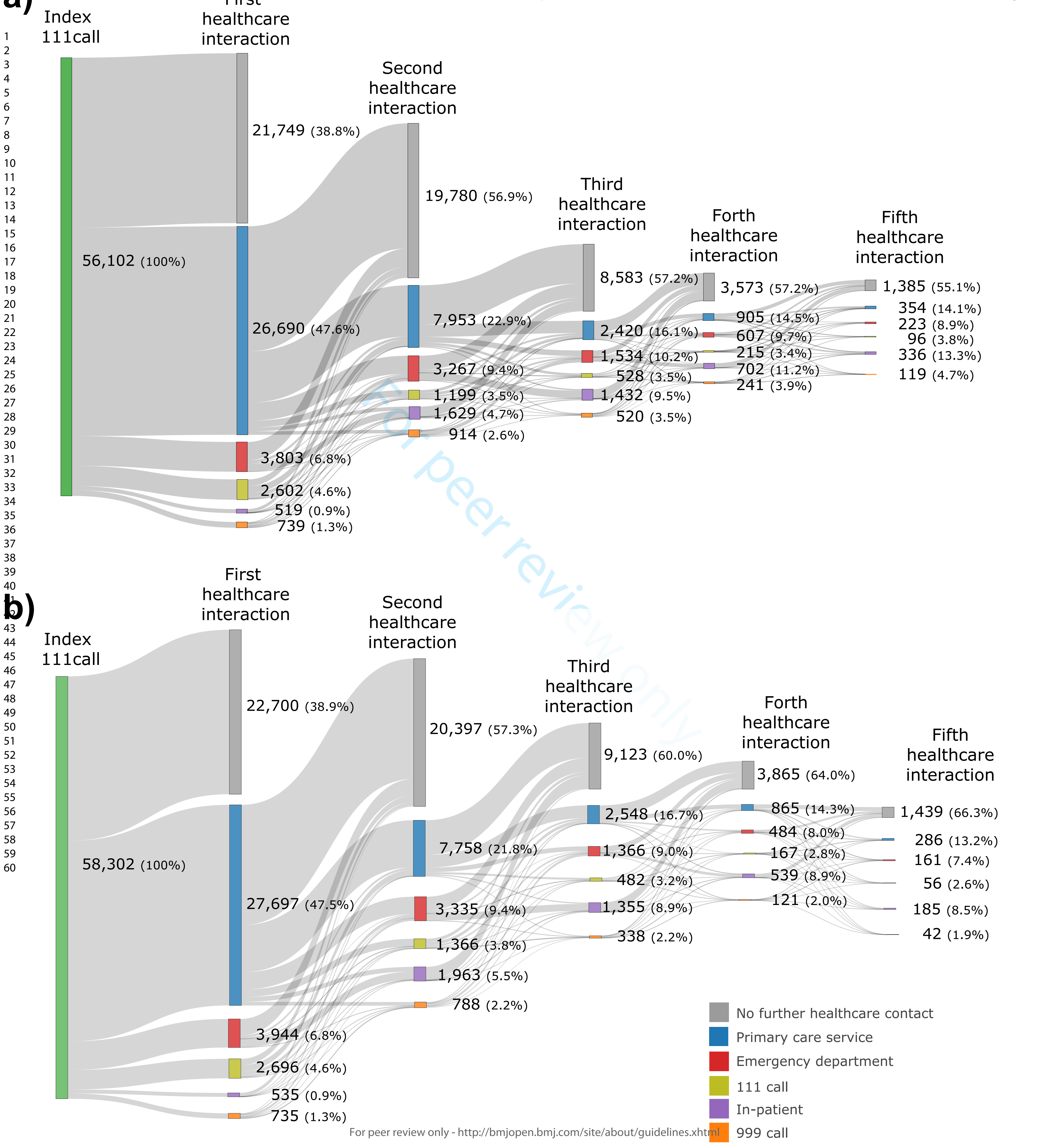

For peer review only

STROBE extension for simulation based research checklist

Item	Item no.	STROBE item	Location	Extension for SBR	Location
Title and abstract	1	a. Indicate the study's design with a commonly used term in the title or the abstract.<br>b. Provide in the abstract an informative and balanced summary of what was done and what was found.	a. Title<br>b. Abstract P2	In abstract or key terms, the MESH or searchable keyword term must have the word simulation or simulated.	Keywords: P1
Introduction					
Background/rationale	2	Explain the scientific background and rationale for the investigation being reported.	P2	Clarify whether simulation is subject of research or investigational method for research.	P3-5
Objectives	3	State specific objectives, including any prespecified hypotheses.	P2		
Methods					
Study design	4	Present key elements of study design early in the paper.	Methods P5–9		
Setting	5	Describe the setting, locations, and relevant dates, including periods of recruitment, exposure, follow-up, and data collection.	Data section P5-6		
Participants	6	a. Cohort study: give the eligibility criteria and the sources and methods of selection of participants. Describe methods of follow-up.<br>b. Cohort study: for matched studies, give matching criteria and number of exposed and unexposed.	a. Data section P5-6<br>b. N/A		
Variables	7	Clearly define all outcomes, exposures, predictors, potential confounders, and effect modifiers.<br>Give diagnostic criteria, if applicable.		Describe the theoretical and/or conceptual rationale for the design of the intervention/exposure.<br>Describe the intervention/exposure with sufficient detail to permit	Methods section: Conceptual modelling DES P5

STROBE extension for simulation based research checklist

				replication.<br>Clearly describe all simulation-specific exposures, potential confounders, and effect modifiers.	
Data sources/ measurement	8	For each variable of interest, give sources of data and details of methods of assessment (measurement).<br>Describe comparability of assessment methods if there is >1 group.	Data section P5-6	In describing the details of methods of assessment, include (when applicable) the setting, instrument, simulator type, timing in relation to the intervention, along with any methods used to enhance the quality of measurements.<br>Provide evidence to support the validity and reliability of assessment tools in this context (if available).	DES Analysis P8
Bias	9	Describe any efforts to address potential sources of bias.	Strengths and weaknesses P13		
Study size	10	Explain how the study size was arrived.	N/A		
Quantitative variables	11	Explain how quantitative variables were handled in the analyses. If applicable, describe which groupings were chosen and why.	Analysis P8-9		
Statistical methods	12	a. Describe all statistical methods, including those used to control for confounding.<br>b. Describe any methods used to examine subgroups and interactions.<br>c. Explain how missing data were addressed.<br>d. Cohort study: if applicable, explain how loss to follow-up was addressed.<br>Case–control study: if applicable, explain how matching of cases and controls was addressed.<br>Cross-sectional study: if applicable, describe	a. Methods P5-9<br>b. Methods P5-9<br>c. N/A<br>d. N/A	Clearly indicate the unit of analysis (e.g., individual, team, system), identify repeated measures on subjects, and describe how these issues were addressed.	Analysis P8

STROBE extension for simulation based research checklist

		analytical methods taking account of sampling strategy.<br>e. Describe any sensitivity analyses.	e. N/A		
Results					
Participants	13	a. Report the numbers of individuals at each stage of the study (e.g., numbers potentially eligible, examined for eligibility, confirmed eligible, included in the study, completing follow-up, and analyzed).<br>b. Give reasons for nonparticipation at each stage.<br>c. Consider use of a flow diagram.	Figure 3		
Descriptive data	14	a. Give characteristics of study participants (e.g., demographic, clinical, social) and information on exposures and potential confounders.<br>b. Indicate the number of participants with missing data for each variable of interest.<br>c. Cohort study: summarize follow-up time (e.g., average and total amount).	Results P9-11	In describing characteristics of study participants, include their previous experience with simulation and other relevant features as related to the intervention(s).	N/A
Outcome data	15	Cohort study: report numbers of outcome events or summary measures over time.<br>Case–control study: report numbers in each exposure category or summary measures of exposure.<br>Cross-sectional study: report numbers of outcome events or summary measures.	Results P9-11		
Main results	16	a. Give unadjusted estimates and, if applicable, confounder-adjusted estimates and their	A. Table 1, P9-10	For assessments involving >1 rater, interrater reliability should be reported.	N/A

STROBE extension for simulation based research checklist

		precision (e.g., 95 % confidence intervals). Make clear which confounders were adjusted for and why they were included.<br>b. Report category boundaries when continuous variables were categorized.<br>c. If relevant, consider translating estimates of relative risk into absolute risk for a meaningful time period.	b. N/A<br>c. N/A		
Other analyses	17	Report other analyses done (e.g., analyses of subgroups and interactions and sensitivity analyses).	Results, P11		
Discussion					
Key results	18	Summarize key results with reference to study objectives.	Discussion P11-12		
Limitations	19	Discuss limitations of the study, taking into account sources of potential bias or imprecision. Discuss both direction and magnitude of any potential bias.	Strengths and weakness P13-14	Specifically discuss the limitations of SBR.	Strengths and weakness P13-14
Interpretation	20	Give a cautious overall interpretation of results considering objectives, limitations, multiplicity of analyses, results from similar studies, and other relevant evidence.	Discussion P11-14		
Generalisability	21	Discuss the generalisability (external validity) of the study results.	Strengths and weaknesses P13-14	Describe generalisability of simulation-based outcomes to patient-based outcomes (if applicable).	Strengths and weaknesses P13-14
Other information					
Funding	22	Give the source of funding and the role of the funders for the present study and, if applicable, for the original study on which the present article is based.	Funding P15	List simulator brand and if conflict of interest for intellectual property exists.	Methods – DES P6-7
